# Enhanced Thermodynamic Stability of UO_2_^2+^ Complex Through Structure Preorganization of N_3_O_2_‐Pentadentate Planar Ligand for Uranium Harvesting from Seawater

**DOI:** 10.1002/advs.202522146

**Published:** 2026-01-18

**Authors:** Ryuto Nabata, Satoru Tsushima, Koichiro Takao

**Affiliations:** ^1^ Laboratory for Zero‐Carbon Energy Institute of Integrated Research, Institute of Science Tokyo 2‐12‐1 N1‐32, O‐okayama Meguro‐ku Tokyo Japan; ^2^ Institute of Resource Ecology Helmholtz‐Zentrum Dresden‐Rossendorf (HZDR) Bautzner Landstraße 400 Dresden Germany

**Keywords:** coordination chemistry, resource development in seawater, selectivity, thermodynamic stability, uranium

## Abstract

Uranium harvesting from seawater is recently attracting special attention as a promising and sustainable resource option of this primary nuclear energy source. Through appropriate preorganization of the ligand backbone, we have achieved a remarkable enhancement in the coordination affinity of an N_3_O_2_‐pentadentate planar ligand with UO_2_^2+^ by more than 5 orders of magnitude compared to bis(2‐hydroxyphenylmethylaminoethyl)amine (H_2_saldian) recognized as the strongest U‐chelator under seawater conditions. 2,6‐Bis(2‐hydroxyphenylmethylaminomethyl)pyridine (H_2_saldamp) was confirmed to form a 1:1 complex, UO_2_(saldamp), in a simulated seawater. Its thermodynamic stability (log *β*_U_ = 33.60 ± 0.01) is superior not only to UO_2_(saldian) (log *β*_U_ = 28.05), but also to any UO_2_^2+^ complexes bearing amidoxime‐based/‐inspired ligands reported so far. H_2_saldamp also exhibits notable separability of UO_2_^2+^ from other concomitant ions, including VO_2_^+^, Al^3+^, Ni^2+^, Cu^2+^, and Zn^2+^ as pronounced by separation factors of >10^12^. While contamination of U by V is one of the issues to be resolved in U harvesting from seawater, their perfect separation can be anticipated using H_2_saldamp due to its negligible affinity for VO_2_^+^. Further preorganization by replacing the H_2_saldian backbone with bis(2‐aminophenyl)amine (H_2_saldiphan) and 1,9‐diaminophenazine (H_2_salphenazine) proved unsuccessful, implying that Lewis‐basicity of all coordinating atoms and equatorial planarity around UO_2_^2+^ must also be taken into consideration.

## Graphical abstract

Thanks to the appropriate structure preorganization of an N_3_O_2_‐pentadentate planar ligand, 2,6‐bis(2‐hydroxyphenylmethylaminomethyl)pyridine (H_2_saldamp) exhibits the strongest coordination affinity to UO_2_^2+^ (log *β*_U_ = 33.60) compared to ligands developed for uranium harvesting from seawater to date, as well as good separability of UO_2_^2+^ from other metal ions concomitantly present in seawater.

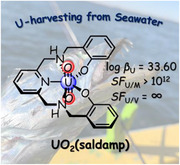

## 1 Introduction

Nuclear power has a much higher energy density compared to other renewable energy sources and does not emit CO_2_, making it an important resource for achieving carbon neutrality. Uranium used in nuclear power generation is currently supplied through mining operations. However, the estimated mineable reserves are expected to last for approximately 100 years [1].

On the other hand, 4.5 billion tons of U are dissolved in seawater, which is about 1000 times greater than its terrestrial stock. The large amount of U in seawater is attracting attention as a sustainable nuclear energy source. However, it is still challenging to practically utilize U from seawater for three primary reasons. First, U in seawater is extremely diluted (∼3.3 ppb = 14 nM). Second, U alone must be selectively collected amongst 78 elements concomitantly present there, some of which are in very high abundance (Na^+^, Cl^–^, Mg^2+^ etc.). Third, UO_2_^2+^ in seawater predominantly forms a very stable triscarbonato complex, [UO_2_(CO_3_)_3_]^4–^, with log *β*_13_ = 21.84 [2]. Therefore, adsorbents with extremely high affinity and selectivity for UO_2_^2+^ is imperative for the viability of U harvesting from seawater.

In the last century, U recovery from seawater had already been a subject of thorough investigations, and the use of resin adsorbents modified with amidoxime groups had been intensively studied [3, 4, 5]. Even today, these amidoximes remain the most frequently utilized functional group worldwide for U recovery from seawater [6, 7, 8, 9, 10]. However, the optimal pH condition for U adsorption using amidoxime groups (pH 4–6) does not perfectly coincide with the condition of actual seawater (pH ∼8) [6, 7, 8, 9, 10]. Moreover, the molecular structures of amidoxime‐based functional groups are not entirely optimized for capturing UO_2_^2+^ from a viewpoint of coordination chemistry [11], because the equatorial plane of UO_2_^2+^ remains largely unsaturated. In recent years, the development of novel ligand designs has emerged as an alternative to amidoxime ligands, such as H_2_BHT, which has been demonstrated to exhibit a strong affinity for UO_2_^2+^ [12]. However, they have not reached to a level of practicality in terms of selectivity for UO_2_^2+^ [6, 7, 8, 9, 10, 12].

**FIGURE 1:**
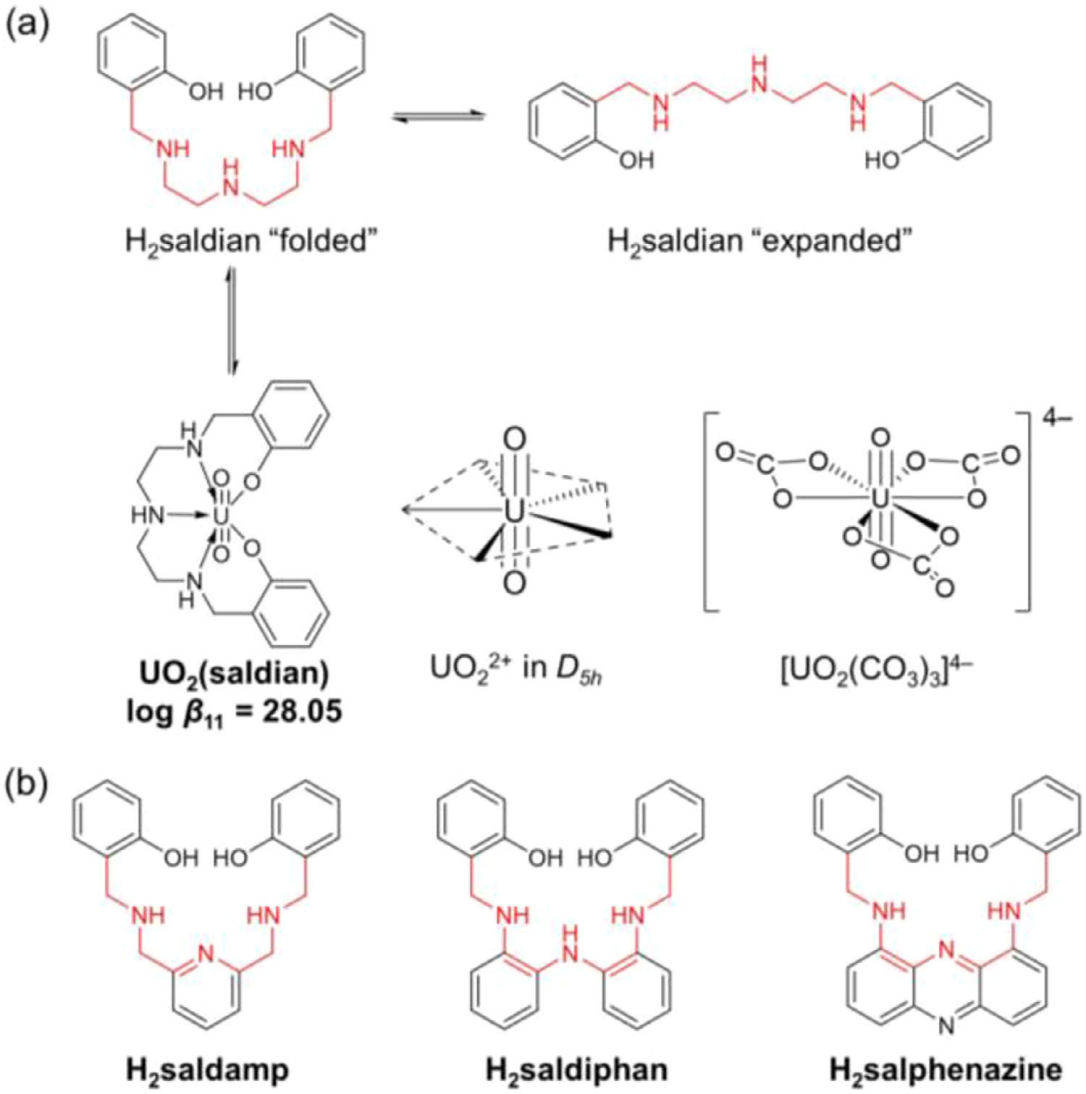
Typical conformations of H_2_saldian in aqueous solution and its complexation with UO_2_^2+^ in seawater together with the representative pentagonal bipyramidal coordination geometry around UO_2_^2+^ in *D*_5_ *_h_* symmetry and [UO_2_(CO_3_)_3_]^4−^ predominantly present in seawater (a), and preorganized N_3_O_2_‐pentadentate planar ligands studied in this work (b).

It has been well‐established that UO_2_^2+^ prefers a planar five‐coordinated structure on its equatorial plane (Figure 1a), a distinct feature of the actinyl series (AnO_2_ *^n^* ^+^; An = U, Np, Pu; *n* = 1, 2) [13]. Accordingly, we have recently developed a fully‐chelating N_3_O_2_‐pentadentate planar ligand, saldian^2−^ (Figure 1a). The thermodynamic stability of its UO_2_^2+^ complex, UO_2_(saldian), (log *β*_U_ = 28.05 ± 0.07) is not only high enough to compete against the formation of [UO_2_(CO_3_)_3_]^4−^, but also reveals its strongest U‐capturing nature compared with any amidoxime‐based or ‐inspired ligands known to date [14].

However, there is still room of improvement with regard to the molecular design of saldian^2−^. As illustrated in Figure 1a, the conformers of H_2_saldian in free form undergo continuous exchange through random rotations around the C−C and C−N bonds, although it is important to note that, in reality, a wider range of variations is likely to be present beyond those depicted in the figure. In contrast, the “folded” conformation must be provided through its complexation with UO_2_^2+^ to form UO_2_(saldian) for U‐capture. Reducing the degree of freedom in the rotational conformations of the ligand molecule can result in a preferred structure for coordination in advance, which would improve the thermodynamic stability of the UO_2_^2+^ complex. Based on this idea, we herein propose improved molecular design of H_2_saldian to provide preorganized N_3_O_2_‐pentadentate planar ligands, namely H_2_saldamp, H_2_saldiphan, and H_2_salphenazine, as listed in Figure 1b. These ligands are believed to exhibit reduced free rotations around the C−C and/or C−N bonds, which would in turn provide preferred orientations for complexation to UO_2_^2+^.

## 2 Results and Discussion

H_2_saldamp was synthesized in 73% yield through a condensation reaction between 2,6‐pyridinecarboxaldehyde and 2‐aminomethylphenol in THF. This reaction afforded a parent Schiff base, which was subsequently reduced through a reductive amination with excess NaBH(OAc)_3_, to convert the azomethine groups (−CH═N−) to their aminomethylene counterparts (−CH_2_−NH−) [14]. The resulting ligand was characterized by ^1^H and ^13^C NMR and IR spectroscopy. Note that H_2_saldamp is also accessible via an alternative synthetic route through the condensation of salicylaldehyde and 2,6‐bis(aminomethyl)pyridine, followed by reductive amination with NaBH(OAc)_3_ in THF in 86% yield.

The protonation/deprotonation equilibria of H_2_saldamp were examined in a 0.50 M NaCl aqueous solution at 298 K through UV‐vis absorption spectrophotometry (Figure S5). The variation of pH resulted in a gradual change in the UV‐vis spectra, and no isosbestic points were identified in this spectral series. This finding suggests the presence of multiple protonation equilibria. The gross protonation constants of saldamp^2−^ (log *β_n_*_H_, *n* = 1–5) were determined by a nonlinear least‐squares fit using the HypSpec program [15] as follows.

(1) $$\mathrm{saldam}p^{2-}+H^{+}=\mathrm{Hsaldam}p^{-}\;\;\log\;\beta_{1H}=14.20$$

(2) $$\mathrm{saldam}p^{2-}+2H^{+}=H_{2}\mathrm{saldamp}\;\;\log\;\beta_{2H}=25.27$$

(3) $$\mathrm{saldam}p^{2-}+3H^{+}=H_{3}\mathrm{saldam}p^{+}\;\;\log\;\beta_{3H}=35.66$$

(4) $$\mathrm{saldam}p^{2-}+4H^{+}=H_{4}\mathrm{saldam}p^{2+}\;\;\log\;\beta_{4H}=44.42$$

(5) $$\mathrm{saldam}p^{2-}+5H^{+}=H_{5}\mathrm{saldam}p^{3+}\;\;\log\;\beta_{5H}=52.10$$

All five‐step protonation equilibria of two phenolic, two amino, and one pyridyl groups were experimentally observed. From the obtained log *β_n_*_H_, the acid dissociation constants, p*K*_a_ *_n_* (*n* = 1–5), were evaluated as p*K*_a1_ = 7.68, p*K*_a2_ = 8.76, p*K*_a3_ = 10.39, p*K*_a4_ = 11.07, and p*K*_a5_ = 14.20. Due to the proton ambiguity arising from the potential tautomerization of 2‐aminomethylphenolic parts, as discussed in previous publications [16, 17, 18], it was not straightforward to correctly assign each p*K*_a_ *_n_* to a specific site in the molecular structure of saldamp^2−^. However, the observed five‐step acid dissociation equilibria should consist of one pyridyl N atom, two amino N atoms, and two phenolic O atoms in a formal chemical sense. p*K_a_*_1_ can be attributed to the deprotonation of the pyridinium NH of H_5_saldamp^3+^. This value is somewhat higher than p*K_a_* of 2,6‐dimethylpyridine (p*K_a_* = 6.77) [19], which constitutes the substructure of H_2_saldamp. It is considered that the proton located at the pyridyl moiety of H_2_saldamp could be stabilized by intramolecular hydrogen bonding. While p*K*_a5_ = 14.20 is somewhat greater than those of ordinary phenols (p*K*_a_ = 7–11) [20], this value would nevertheless be acceptable because deprotonation from monoanionic Hsaldamp^−^ is more difficult than electrically neutral H_2_saldamp, having p*K*_a4_ = 11.07. It could be hypothesized that the phenolic H^+^ is stabilized by an intramolecular hydrogen bond between the O−H and N of the amino or pyridyl moieties. The remaining p*K*_a2_ and p*K*_a3_ values are reasonably assigned to the secondary amino groups of saldamp^2−^ having a similar molecular skeleton to saldian^2–^ we reported previously [14].

**FIGURE 2:**
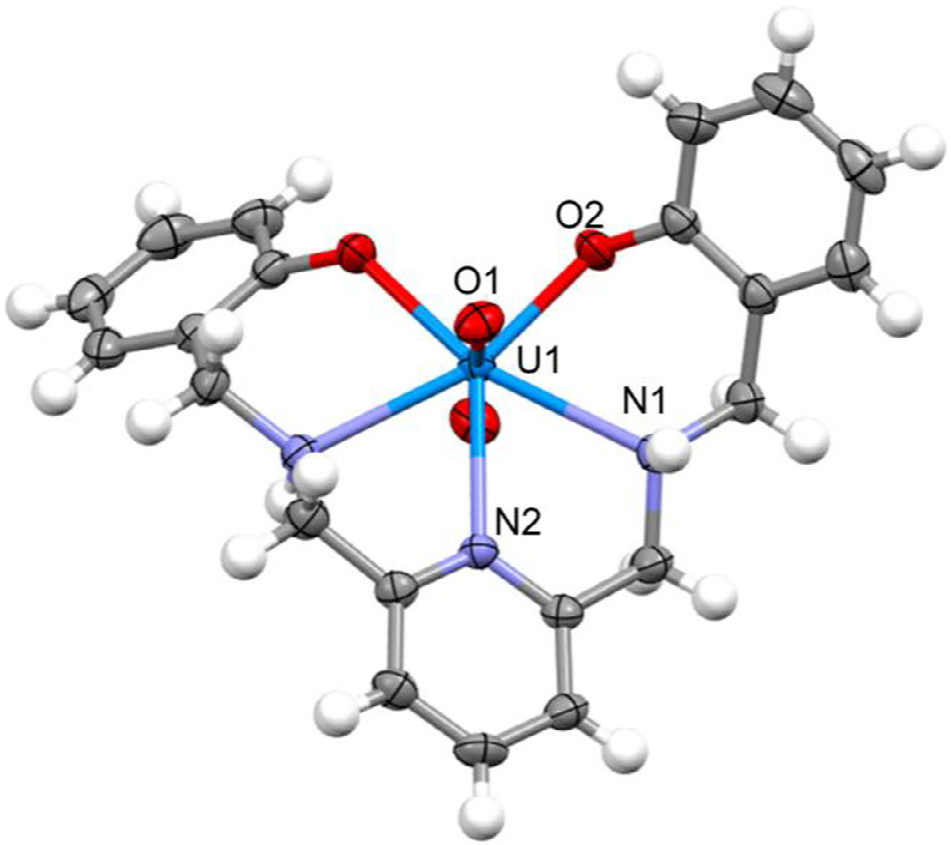
Molecular structure of UO_2_(saldamp) at 50% probability level. Selected structural parameters: U1−O1 1.787(2) Å, U1−O2 2.225(2) Å, U1−N1 2.616(2) Å, U1−N2 2.591(3) Å, O1−U1−O1^i^ 174.53(13)°, O2−U1−O2^i^ 88.54(11)°,O2−U1−N1 73.61(9)°, N1−U1−N2 62.14(5)° (symmetry operation: (i) 1−*x*, *y*, 1/2−*z*).

A UO_2_(saldamp) complex was successfully prepared as reddish crystalline powder in 46% yield from a mixture of H_2_saldamp and UO_2_(NO_3_)_2_·6H_2_O in THF, and was characterized by ^1^H NMR, IR, and X‐ray crystallography (Figures S1–S4). Figure 2 shows the molecular structure of UO_2_(saldamp) recrystallized from DMSO and water. As a result, the formation of the anticipated UO_2_^2+^ complex featuring a predicted planar five‐coordinated structure was successfully confirmed. The UO_2_^2+^ moiety exhibits typical structural features of this class of complexes in terms of bond lengths between U and axial O (U1−O1: 1.787(2) Å) and slightly bent, but still close to linear, O≡U≡O unit (∠O1−U1−O1^i^ = 174.53(13)°, symmetry operation: (i) 1−*x*, *y*, 1/2−*z*). The distance between U1 and phenolic O2 (2.225(2) Å) is consistent with those in UO_2_(saldian) (2.215−2.230 Å, Table S1) and in other related complexes (2.224−2.300 Å) reported so far [21]. The U1−N1 and U1−N2 distances (2.616(2), 2.591(3) Å) are also similar to those in UO_2_(saldian) (U1−N1 = 2.613‐2.650, U1−N2 = 2.574(3) Å, Table S1) and corresponding Schiff base complexes, UO_2_(saldien) (2.586−2.602, 2.581 Å) [22]. One of the decisive differences between UO_2_(saldamp) and its related UO_2_^2+^ complexes is that the coordinating atoms and U1 all exist on the same plane. Indeed, the sum of the bite angles of these chelate rings plus O2−U1−O2^i^ (symmetry operation: (i) 1−*x*, *y*, 1/2−*z*) is found to be 360.06 ± 0.07°, showing the near‐perfect planarity of the UO_2_^2+^ equatorial coordination. Such high degree of flatness has never been achieved, even in UO_2_(saldian) (362.96 ± 0.08°) we developed previously for the same purpose [14]. Therefore, saldamp^2−^ may offer optimal coordination to UO_2_^2+^ compared with saldian^2−^ and other ligands designed for U recovery from seawater despite structural constraints imposed by the introduction of a rigid pyridyl moiety. Furthermore, the symmetry of UO_2_(saldamp) is greater than that of UO_2_(saldian) as evidenced by the presence of a *C*_2_ axis through U1 and N2 only in the former system. This observation is consistent with the higher stability of UO_2_(saldamp) (vide infra).

**FIGURE 3:**
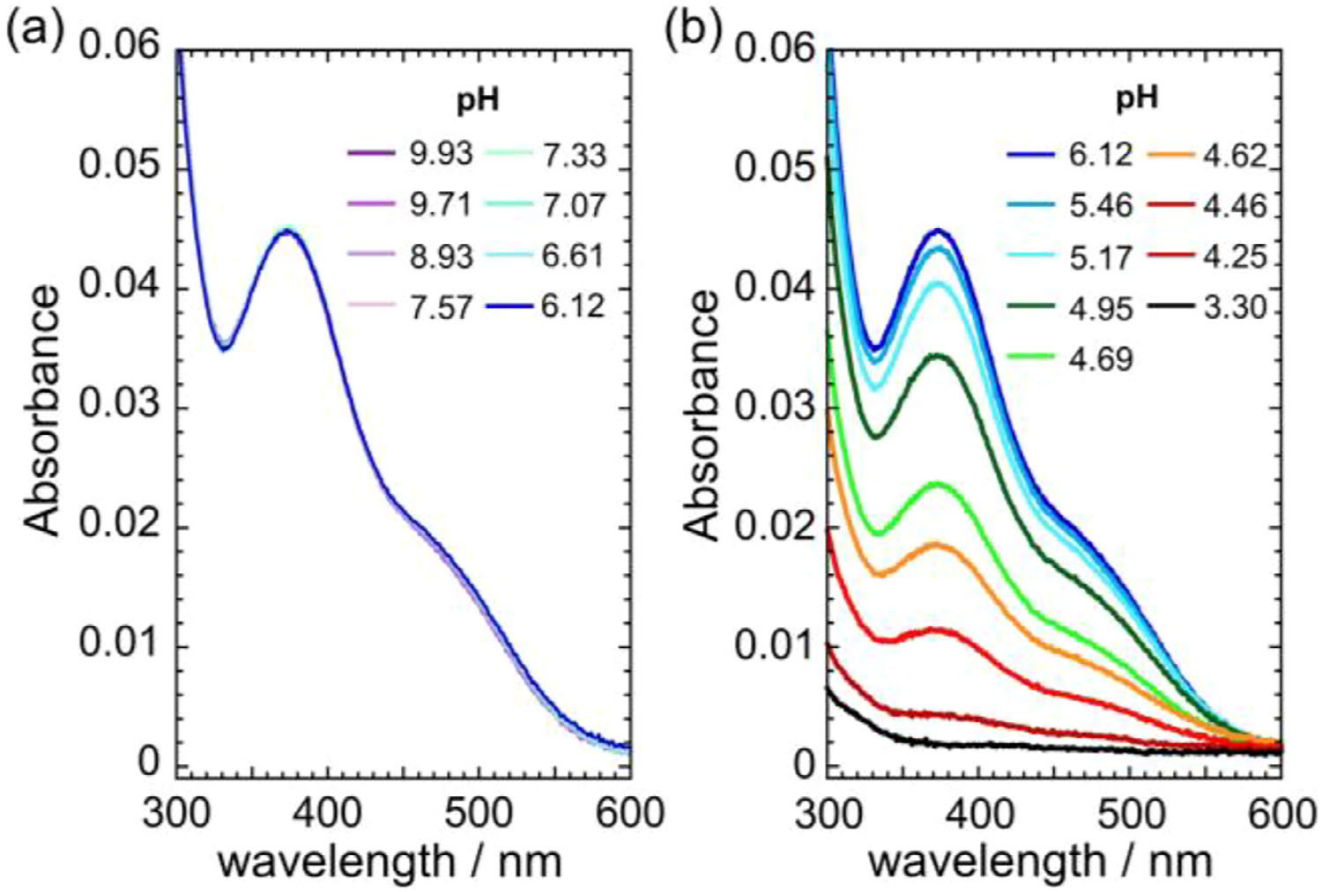
UV–vis absorption spectra of UO_2_(saldamp) (2.0 × 10^−5^ M) at pH 9.93‐6.12 (a), and pH 6.12‐3.30 (b) in 0.50 M NaCl aqueous solution at 298 K.

The complexation of saldamp^2−^ with UO_2_^2+^ in 0.50 M NaCl(aq) was investigated by spectrophotometric titration at 298 K. In order to simulate the conditions of seawater, such experiments should be conducted under CO_3_^2−^‐abundant conditions, namely 2.3 mM HCO_3_^−^/CO_3_^2−^, as previously employed for our study of saldian^2−^ [14]. On the other hand, when the same approach was employed for the current UO_2_^2+^‐saldamp^2−^ system, the determination of the stability constant of UO_2_(saldamp) was unsuccessful. This is because the formation of UO_2_(saldamp) was too preparative to analyze the complexation equilibrium of interest (Figure S7). Therefore, the chemistry in question was studied by means of UV–vis titration under pH variation, where HCO_3_^−^/CO_3_^2−^ were excluded due to the necessity of acidifying the system of interest in which the carbonates may escape as CO_2_(g). Figure 3 shows a series of UV–vis spectra with pH variation. At the initial condition of pH 9.93 (Figure 3a), an absorption band and shoulder were observed at 380 and 480 nm, respectively. The observed spectroscopic trend indicates that UO_2_(saldamp) is formed as shown in Equation (6), because neither H_2_saldamp and any of its protonated/deprotonated species (Figure S5) nor UO_2_^2+^ exhibit such a spectral pattern.

(6) $$U{O_{2}}^{2+}+\mathrm{saldam}p^{2-}⇄UO_{2}\left(\mathrm{saldamp}\right)$$

It is noteworthy that no observable differences have been detected between pH 9.93 and 6.12, and that these spectra are identical to that recorded under presence of 2.3 mM HCO_3_^−^/CO_3_^2−^ (Figure S7), indicating that UO_2_(saldamp) is predominantly formed within this pH range, including in the context of seawater conditions (pH ∼ 8). As the pH level decreased from 6.12 to 3.30 (Figure 3b), the intensity of these characteristic absorption bands gradually diminished, implying that UO_2_^2+^ was released from saldamp^2−^. Indeed, H_5_saldamp^3+^ occurring predominantly at pH 3.30 (see Figure S6) exhibits minimal light absorption in the wavelength region displayed in Figure 3.

The UV‐vis absorption spectra of the UO_2_^2+^‐saldamp system unequivocally demonstrate the occurrence of complex formation between UO_2_^2+^ and saldamp^2–^ ligand. This assertion is corroborated by the presence of absorption features within the 350–550 nm region (Figure 3). Preliminary observations indicate that the absorption in question is situated within the “fingerprint region” of uranyl(VI) absorption, as evidenced by the energy range (350–550 nm) of the observed features. The absorption in the fingerprint region is attributed to the axial O (O_yl_) to U LMCT with vibronic progression. Time‐dependent density functional theory (TD‐DFT) calculations were performed on the UO_2_(saldamp) complex, revealing relatively strong absorption features in the 450 to 500 nm region (Figure S8). A thorough examination reveals that these electronic transitions occur via ligand to uranium LMCT, and not O_yl_ to U LMCT as was initially hypothesized. The ligand to uranium LMCT is typically observed in the UV region (200‐350 nm) for uranyl(VI) aquo complexes, and is commonly structureless and non‐specific [23]. In the case of the UO_2_(saldamp) complex, the absorption band exhibits significant redshift toward the visible range, accompanied by the presence of specific absorption features. This phenomenon can be attributed to the enhanced rigidity of the specific binding of saldamp^2–^ in comparison to that of aquo ligand.

In order to estimate the stability constant of UO_2_(saldamp) under simulated seawater conditions (0.50 M NaCl(aq)), the spectral series of Figure 3 was analyzed using HypSpec (Figure S9) [15]. As a result, the logarithmic gross stability constant of UO_2_(saldamp) (log *β*_M_ = log{[M(saldamp)]/[M][saldamp^2−^]}, M = UO_2_^2+^) was determined to be 33.60 ± 0.01. In the previous study, we have designed saldian^2−^ ligand and reported it as a strongest UO_2_^2+^‐capturer having log *β*_U_ = 28.05 [14]. The thermodynamic stability of UO_2_(saldamp) is more than five orders of magnitude greater than that of UO_2_(saldian), and therefore, saldamp^2−^ updated the record of log *β*_U_ of ligands developed for U harvesting from seawater. The enhanced affinity of saldamp^2−^ for UO_2_^2+^ can be attributed to the structural preorganization of its ligand skeleton. This is evidenced by the substitution of the center −CH_2_−NH−CH_2_− moiety of saldian^2−^ with the pyridyl group of saldamp^2−^ as illustrated in Figure 1, where all sp^3^ C/N atoms in the former have been replaced by the sp^2^ ones in the latter. As discussed above, lesser structural constraints between U1 and the coordinating atoms in UO_2_(saldamp) is evident from the perfect planarity of its equatorial coordination. This contributes to the enhanced affinity of saldamp^2−^ for UO_2_^2+^. The observed difference in log *β*_U_ between UO_2_(saldamp) and UO_2_(saldian) indicates that the former is more stabilized by 7.5 kcal·mol^−1^ than the latter, which somewhat exceeds sum of the typical energy barriers of C−N bond rotation in secondary amines (2 × 3.0‐3.2 kcal·mol^−1^ = 6.0‐6.4 kcal·mol^−1^) [24, 25, 26]. Therefore, the enhanced stability of the UO_2_^2+^ complex with saldamp^2−^ must be also attributable to the factors other than a reduced degree of freedom for rotational conformation. This can be attributed to the reduced steric hindrance around the pyridyl N atom in the equatorial plane of UO_2_^2+^ compared with the secondary amino group. In fact, pyridine‐type ligands generally exhibit stronger coordination to metal ions, such as Ru^2+^ [27], Pd^2+^ [28], and Co^2+^[29], compared with secondary amines because of their smaller steric demands demonstrated by cone angles (*θ* = 91.9° for pyridine and 4‐methylpyridine versus 119° for NHMe_2_, 125° for NHEt_2_) [30]. While π back‐bonding was also identified as an additional factor contributing to the enhanced stability of the Ru^2+^‐pyridine coordination, [27] such a bonding manner cannot be anticipated in the present system of UO_2_(saldamp) with U formally taking a 5f^0^ closed‐shell electronic configuration.

**FIGURE 4:**
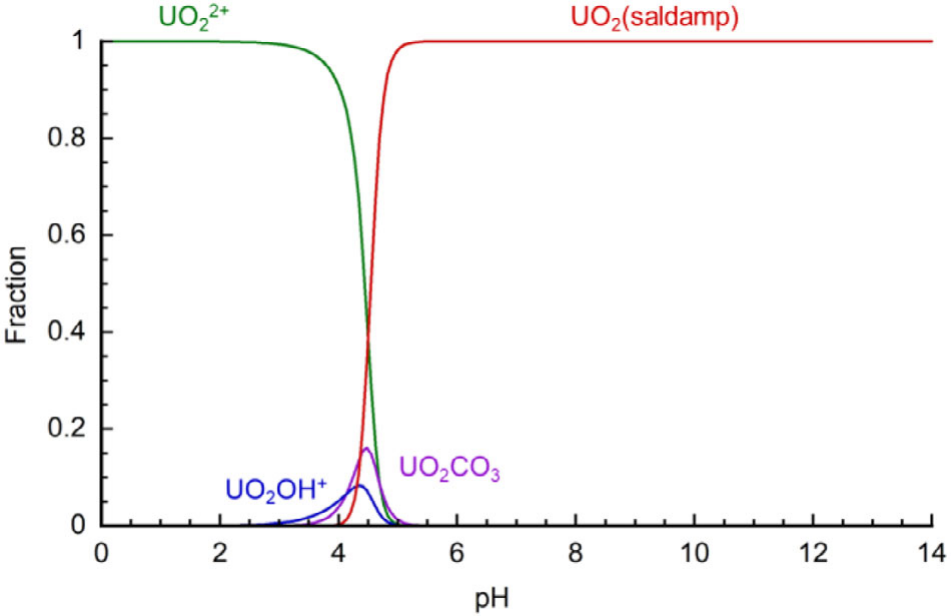
Speciation diagram of UO_2_^2+^ (3.3 ppb = 14 nM) with saldamp^2−^ (0.1 mM) at 0.5 M NaCl + 2.3 mM HCO_3_^−^/CO_3_^2−^ and 298 K.

Needless to say, it is evident that the current log *β*_U_ of UO_2_(saldamp) is incomparably higher than those of any known amidoxime‐based or ‐inspired ligands and other molecules designed for U recovery from seawater to date (e.g., log *β*_U_: 13.6 (acetamidoximate) [31], 16.5 (1,10‐phenanthroline‐2,9‐dicarboxylate) [31], 17.8 (glutarimidedioximate) [32], 17.47 (2,6‐bis[hydroxy(methyl)‐amino]‐4‐morpholino‐1,3,5‐triazinate) [12]. Additionally, the thermodynamic stability of UO_2_(saldamp) is even superior to those of N_4_O_2_‐hexadentate hox^2−^ (log *β*_U_: 26.43) and CHXhox^2−^ (log *β*_U_: 26.7) recently developed as ^230^U‐chelators for radiopharmaceuticals [33]. Indeed, the calculated speciation diagram of UO_2_^2+^ (3.3 ppb = 14 nM in total) under the seawater condition (0.5 M NaCl + 2.3 mM HCO_3_^−^/CO_3_^2−^) as a function of pH under the presence of 0.1 mM saldamp^2−^ (Figure 4) reveals that UO_2_(saldamp) is predominantly formed in a broad pH > 5, which includes pH 8 of the typical seawater condition. This species distribution also implies that, once captured by saldamp^2−^ in seawater, UO_2_^2+^ can be readily released through the acidification of the aqueous eluent below pH 4. This indicates that the adsorption/desorption cycles of UO_2_^2+^ for collecting this nuclear energy source from seawater will be facilitated by pH control.

To assess the specific selectivity of saldamp^2−^ toward UO_2_^2+^, we also measured the stability constants of other metal ions (Ms) coexisting in seawater. Al^3+^, Ni^2+^, Cu^2+^, Zn^2+^, and VO_2_^+^ were selected based on their abundances in seawater and their capacity to form complex with saldian^2−^ we studied previously [14]. Consequently, most of the examined Ms exhibited spectral changes due to complexation (Figure S10). The estimated log *β*_M_ values of the M‐saldamp^2−^ complexes are summarized in Table 1, along with their separation factors (*SF*_U/M_) and their effective *SF*_U/M_ (*SF*_U/M_^eff^ = *SF*_U/M_([UO_2_^2+^]_seawater_/[M*^n^* ^+^]_seawater_)) for UO_2_^2+^ in comparison to the specific Ms. log *β*_M_ values of the complexes of these Ms with saldamp^2−^ varied by several orders of magnitude compared to those with saldian^2−^. However, UO_2_^2+^ still exhibits the largest positive variation in log *β*_U_ through the transition from saldian^2−^ to saldamp^2−^. As a result, the *SF*_U/M_ values of saldamp^2−^ have been improved by at least four orders of magnitude (for Cu^2+^), and up to ten orders of magnitude (for Al^3+^). These results indicate that saldamp^2−^ exclusively captures UO_2_^2+^ in seawater. It is worth noting that the observed spectrum at each [VO_2_^+^]/[saldamp^2−^] ratio appears to consist only of the superposition of these components at different mixing ratios, as shown in Figure S10f. This implies that H_2_saldamp exhibits virtually no affinity with VO_2_^+^. This critically differs from other previously developed ligands for U harvesting from seawater, where UO_2_^2+^ complexation or adsorption always suffered from competing VO_2_^+^. Using saldamp^2−^ allows for the perfect separation of UO_2_^2+^ from VO_2_^+^, which was previously unattainable, even with saldian^2−^ [14].

**TABLE 1:** Logarithmic Gross Stability Constants (log *β*_M_) of Various Metal Ions (Ms) with saldamp^2−^ and saldian^2−^ under Simulated Seawater Condition (0.50 M NaCl, 2.3 mM HCO_3_^−^/CO_3_^2−^, 298 K) together with Separation Factors (*SF*_U/M_ = *β*_U_ / *β*_M_) for UO_2_^2+^ Compared with Specific M.

M	molarity (mol·L^−1^) in seawater*^a^*	saldamp^2−^ log *β*_M_	*SF*_U/M_	*SF*_U/M_^eff^ *^b^*	saldian^2−^ log *β*_M_	*SF*_U/M_	*SF*_U/M_^eff^
UO_2_^2+^	1.4 × 10^−8^	33.60 ± 0.01	−	−	28.05 ± 0.07	−	−
VO_2_^+^	3.7 × 10^−8^	n.a.*^c^*	n.a.*^c^*	n.a.*^c^*	24.79 ± 0.02	1.8 × 10^3^	690
Al^3+^	3.7 × 10^−8^	19.61 ± 0.02	9.8 × 10^13^	3.7 × 10^13^	24.35 ± 0.01	5.0 × 10^3^	1.9 × 10^3^
Ni^2+^	1.1 × 10^−7^	17.20 ± 0.03	2.5 × 10^16^	3.2 × 10^15^	20.58 ± 0.03	3.0 × 10^7^	3.8 × 10^6^
Cu^2+^	1.4 × 10^−8^	21.24 ± 0.02	2.3 × 10^12^	2.3 × 10^12^	19.73 ± 0.01	2.1 × 10^8^	2.1 × 10^8^
Zn^2+^	7.7 × 10^−8^	19.72 ± 0.09	1.9 × 10^14^	3.5 × 10^13^	18.20 ± 0.01	7.1 × 10^9^	1.3 × 10^10^
ref.		This work			[14]		

^a^ Molarity (M = mol·dm^−3^) in seawater taken from Ref. [34].

^b^ Effective *SF*_U/M_, *SF*_U/M_^eff^ = *SF*_U/M_([UO_2_^2+^]_seawater_/[M*^n^* ^+^]_seawater_).

^c^ Not available due to negligible complexation under tested conditions.

**FIGURE 5:**
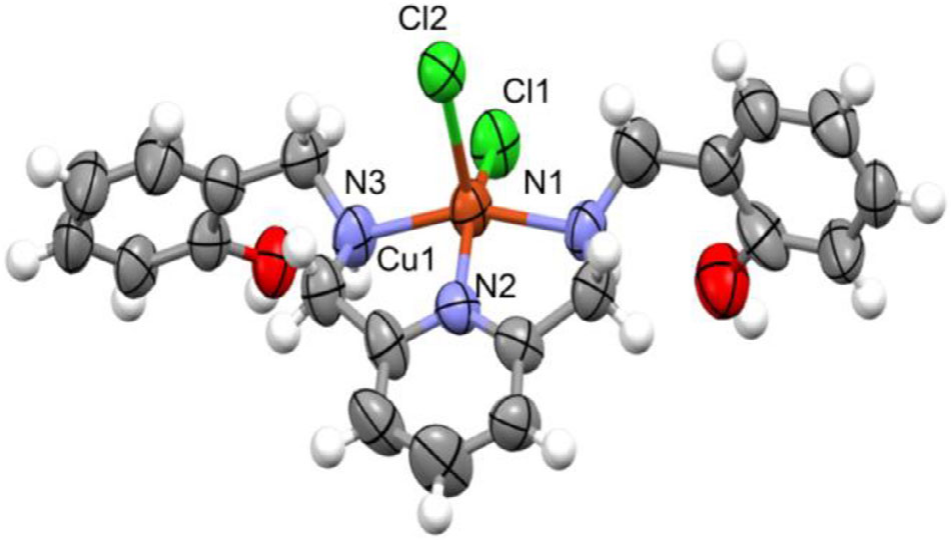
Molecular structure of CuCl_2_(H_2_saldamp) at 50% probability level. Selected structural parameters: Cu1–N1 2.504(9) Å, Cu1–N2 1.932(7) Å, Cu1–N3 2.061(9) Å, Cu1–Cl1 2.224(2) Å, Cu1–Cl2 2.608(3) Å, N1–Cu1–N2 81.2(3)°, N2–Cu1–N3 82.1(3)°,N3–Cu1–Cl1 96.0(2)°, Cl1–Cu1–N1 95.1(2)°.

The enhanced selectivity exhibited by saldamp^2−^ for UO_2_^2+^, especially its perfect separability of UO_2_^2+^ from VO_2_^+^, is attributable to the moderate rigidity and planarity of the ligand structure associated with the pyridyl moiety, which has been shown to suppress the possibility of rotational motions of the ligand in comparison to saldian^2−^. Consequently, other Ms exhibited reduced improvements in their affinity, whereas UO_2_^2+^ demonstrated drastic stabilization of its complexation. To verify this hypothesis, we have attempted to prepare saldamp^2−^ complexes of these Ms. As a result, single crystals of CuCl_2_(H_2_saldamp) were obtained. The X‐ray crystallography of this Cu(II) complex (Figure 5) revealed that only three N atoms of H_2_saldamp are involved in chelation with Cu^2+^, while two Cl^−^ are also part of its coordination sphere, to form a square pyramidal structure of CuCl_2_(H_2_saldamp). Interestingly, the phenolic OH groups remain protonated and are precluded from complex formation with Cu^2+^. While the Cu−N bond distances ranging from 1.93 to 2.06 Å are actually shorter than the U−N distances found in UO_2_(saldamp) (Figure 2), the stability of Cu(II) complex is lower than that of UO_2_(saldamp) due to the weaker chelating effect associated with the reduced number of chelate rings. This situation may also apply to other Ms, although any attempts to crystallize their complexes with H_2_saldamp were unsuccessful thus far.

**FIGURE 6:**
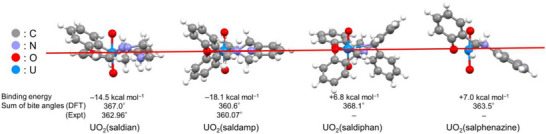
Side views of the DFT‐optimized structures of UO_2_(saldian), UO_2_(saldamp), UO_2_(saldiphan), and UO_2_(salphenazine). Red line: mean plane of equatorial coordination around U.

As demonstrated above, we are now aware that the coordination strength of a N_3_O_2_‐pentadentate planar ligand and its selectivity toward UO_2_^2+^ can be enhanced by preorganizing its coordinating atoms. We therefore tested two additional ligands with further decreased degree of freedom in C−C and/or C−N bond rotations, namely H_2_saldiphan and H_2_salphenazine, as illustrated in Figure 1b. Contrary to our expectations, neither saldiphan^2−^ nor salphenazine^2−^ formed five‐coordinated complexes with UO_2_^2+^. However, these results are reasonable in the usual chemical sense. When H_2_saldiphan was employed, the H atom of its central amine was oriented toward the inside of the “folded” structure of the molecule due to the sp^2^ nature of the parent N atom. As a result, coordination of saldiphan^2−^ with UO_2_^2+^ is inhibited. Moreover, it is difficult to maintain the planarity of saldiphan^2−^ in the “folded” form due to steric hindrance between the H atoms of the neighboring *o*‐phenylene diamine moieties, as evidenced by the free form of bis(2‐aminophenyl)amine and its Ni(II) complex reported elsewhere (Figure S11) [35, 36]. When H_2_salphenazine was tested, a similar problem persisted. The sp^2^ nature of the NH groups directly connected to the phenyl rings in salphenazine^2−^ disturbs its coordination with UO_2_^2+^. A set of these unsuccessful cases imply that Lewis‐basicity of all coordinating atoms oriented to the metal‐center must be guaranteed in the molecular design of N_3_O_2_‐pentadentate planar ligand for U harvesting from seawater. At this stage, we are interested in theoretically verifying experimental trend as well as supporting our hypothesis regarding structural planarity, using quantum chemical calculations. Density functional theory (DFT) calculations at the B3LYP level including dispersion corrections were performed on four set of ligands, namely saldian^2−^, saldamp^2−^, saldiphan^2−^, and salphenazine^2−^. The structures of the protonated free ligands (H_2_L) as well as those bound to UO_2_^2+^ (UO_2_(L)) were optimized by DFT calculations, and the Gibbs binding energy has been estimated. As a result, the UO_2_^2+^ binding energy of each ligand was calculated to be −14.5, −18.1, +6.8, and +7.0 kcal mol^−1^ for saldian^2−^, saldamp^2−^, saldiphan^2−^, and salphenazine^2−^, respectively. The calculated Gibbs energy well‐reproduced experimental trend that showed saldamp^2−^ as the strongest UO_2_^2+^‐binder, whereas saldiphan^2−^ and salphenazine^2−^ showing least (or no) affinity. The energetic cost of UO_2_^2+^ binding for each ligand was additionally calculated by determining the energetic cost of their conformational change. The saldamp^2−^ requires 5.0 kcal mol^−1^ less energetic cost than saldian^2−^ for coordinating UO_2_^2+^, thereby supporting our hypothesis that optimal structural rigidity facilitates the binding process. The calculations also confirmed the structural features of the UO_2_^2+^ complex that we discussed already. In Figure 6, the “side views” of four UO_2_^2+^ complexes studied here are summarized together with the calculated binding energies and sum of bond angles in the UO_2_^2+^ equatorial plane. Consequently, it reveals UO_2_(saldamp) being the only complex showing near‐perfect planarity in its equatorial shell, whereas other three complexes showing significant deviations. The O_yl_≡U−N2 angle is 86.6° in UO_2_(saldamp), whereas it is 79.9° in UO_2_(saldian), implying that the steric hindrance of the coordinating atoms of saldamp^2−^ with O_yl_ of UO_2_^2+^ should be smaller than those of saldian^2−^ to gain the enhanced thermodynamic stability.

## 3 Conclusion

In conclusion, we have designed and synthesized new preorganized N_3_O_2_‐pentadentate ligands with planar sp^2^ pyridyl backbone. It was revealed that saldamp^2−^ exhibits the strongest affinity for UO_2_^2+^ (log *β*_U_ = 33.60) among the ligands that were designed for U recovery from seawater to date. This result was also supported by DFT calculations. The remarkably high affinity for UO_2_^2+^ can be attributed to the preorganization of the ligand backbone and the enhancement of the planarity of the coordinating atoms located in the equatorial plane. Furthermore, the study confirmed an extremely high selectivity for UO_2_^2+^ (*SF*_U/M_ > 10^12^). This is particularly evident in the context of VO_2_^+^, which has otherwise proven to be a formidable challenge in terms of its removal from UO_2_^2+^ within existing technological capabilities. The optimal separation of UO_2_^2+^ from VO_2_^+^ was made possible by the absence of any affinity exhibited by saldamp^2−^ for VO_2_^+^. On the other hand, saldiphan^2−^ and salphenazine^2−^ cannot coordinate effectively with UO_2_^2+^. Therefore, these cases suggested that, in addition to reducing the freedom of C−C and/or C−N bond rotations by preorganization of the ligand structure, localized Lewis basicity of all coordinating atoms with proper orientations to capture UO_2_^2+^ and equatorial planarity of a formed UO_2_^2+^ complex also have to be optimized to enhance the thermodynamic stability of a UO_2_^2+^ complex. To socially implement U harvesting from seawater, saldamp^2–^ skeleton must be chemically installed onto a polymer chain. For example, it will be realized by S_N_2 reaction of one of the secondary amino groups of H_2_saldamp to chloromethylated polystyrene as mentioned in our former work [14]. All the details of chemical experiments and data analyses as well as theoretical calculations were described in Supporting Information together with additional references cited therein [37, 38, 39, 40, 41, 42, 43, 44, 45, 46, 47, 48, 49, 50, 51, 52, 53, 54, 55].

## Conflicts of Interest

The author declare no conflict of interests

## Supporting information




**Supporting File 1**: advs73779‐sup‐0001‐SuppMat.pdf.

**Supporting File 2**: advs73779‐sup‐0002‐Data.zip.

## Data Availability

The data that support the findings of this study are available from the corresponding author upon reasonable request.
