# Supplementary material for: Enhanced Thermodynamic Stability of UO2 2+ Complex Through Structure Preorganization of N3O2‐Pentadentate Planar Ligand for Uranium Harvesting from Seawater
Source: Adv Sci (Weinh). 2026 Jan 18;13(17):e22146. doi: 10.1002/advs.202522146 (PMC13042446; doi:10.1002/advs.202522146)
Supplement: Supplementary file 1 — Supporting File 1: advs73779‐sup‐0001‐SuppMat.pdf. [file ADVS-13-e22146-s001.pdf]

Supporting Information

Table of Contents.

		page
**Experimental Procedure**		S3
**Figure S1.**	^{1}H NMR spectra of H_2saldamp (black) and UO_2(saldamp) (blue) in DMSO-d_6.	S9
**Figure S2.**	Molecular structure of UO_2(saldamp) and Newman projections along C−N bonds of UO_2(saldamp). Red : *endo* H, Blue : *exo* H.	S9
**Figure S3.**	^{13}C NMR spectrum of UO_2(saldamp) in DMSO-d_6.	S10
**Figure S4.**	IR spectrum of UO_2(saldamp).	S10
**Figure S5.**	UV-vis absorption spectra of H_2saldamp (1.0 × 10^{-4} M) at different pH in 0.50 M NaCl + 2.3 mM HCO_3^-/CO_3^{2-} aqueous solution at 298 K.	S13
**Figure S6.**	Speciation diagram of saldamp^{2-} species (0.10 mM) at 0.50 M NaCl + 2.3 mM HCO_3^-/CO_3^{2-} and 298 K.	S13
**Table S1.**	Selected bond lengths (Å) of UO_2(saldamp) and UO_2(saldian).	S14
**Figure S7.**	(a) UV-vis absorption spectra of aqueous solution of H_2saldamp (1.0×10^{-4} M) at different total UO_2^{2+} concentrations under the simulated seawater condition (0.50 M NaCl +2.3 mM HCO_3^-/CO_3^{2-}, pH 8, 298 K) and (b) absorbance at 380 nm in panel (a).	S14
**Figure S8.**	Predicted vertical transitions (vertical black lines) of the UO_2(saldamp) complex and the key excited states and molecular orbitals (MO 114 to MO118) involved.	S15
**Figure S9.**	A screenshot of the HypSpec analysis of log β_U of UO_2(saldamp).	S15
**Figure S10.**	UV-vis absorption spectra of aqueous solutions of H_2saldamp (1.0 × 10^{-4} M) at different total concentrations of selected metal ions (M's) under the simulated seawater condition (0.50 M NaCl + 2.3 mM HCO_3^- /CO_3^{2-}, pH 8, 298 K). M = Al^{3+} (a), Ni^{2+} (b), Cu^{2+} (c), Zn^{2+} (d), and VO_2^+ (e). Blue lines indicate original absorption spectra of M's at 0.1 mM, if there is significant absorption. (f) Absorbance at 270 nm in panel (e) (red line) together with sum of the absorbance of VO_2^+ and saldamp^{2-} at a specified [VO_2^+]/[saldamp^{2-}] (blue line). Error bars represent estimated experimental errors based on uncertainty in actual pipetting operations.	S16
**Figure S11.**	Molecular structures of free form of bis(2-aminophenyl)amine (a), its Ni(II) complex (b) reported elsewhere.	S17
**Cartesian Coordinates of DFT-Optimized Structures**		S18

Reference S24

Experimental Procedure

Syntheses of Pentadentate Planar Ligands.

H_2saldamp.

Method (a): 2-Aminomethylphenol (1.01 g, 8.20 mmol, Tokyo Chemical Industry Co., Ltd) were added to a THF(30 mL) solution dissolving 2,6-pyridinedicarboxaldehyde (552 mg, 4.01 mmol, Tokyo Chemical Industry Co., Ltd) under vigorous stirring at 0 °C for 30 min, and then $NaBH(OAc)_3$ (2.58 g, 12.2 mmol, Wako Pure Chemical Industries, Ltd.) were loaded portionwise. The yellow suspension was stirred at 0 °C for 30 min, and then further agitated at RT for 2 h. Water was poured into the nearly colorless suspension to quench the reaction, followed by additional stirring for 15 min. After removal of THF by evaporation, 7.2 M NaOH aq was dropwise added to this aqueous mixture to make its pH 8-9, where oily layer was separated from the aqueous phase. This oily material was extracted by CH_2Cl_2 (50 mL), washed with a based brine (50 mL), and dried over $MgSO_4$ (Wako Pure Chemical Industries, Ltd.). This filtrate was evaporated to obtain a pale-yellow oil (1.04 g, 2.97 mmol, 74% yield).

^{1}H NMR (δ/ppm vs. TMS, $CDCl_3$) 7.62 (t, 1H, Py−H), 7.18 (td, 2H, Ph−H), 7.11 (d, 2H, Py−H), 6.97 (dd, 2H, Ph−H), 6.84 (dd, 2H, Ph−H), 6.77 (dd, 2H, Ph−H), 4.01 (s, 4H, Py−CH_2−N), 3.94 (s, 4H, N−CH_2−Ph). ^{13}C NMR (δ/ppm vs. TMS, $CDCl_3$) 158.20, 157.65, 137.26, 128.69, 128.43, 122.42, 121.30, 119.02, 116.42, 52.92, 51.92. IR (v/cm^{-1} diamond ATR) 1086 ($\nu_{C\text{-}N}$).

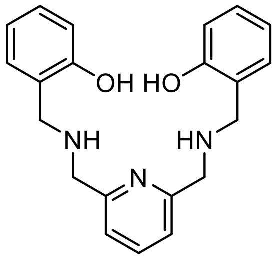

H_2saldamp

Method (b):

P1.[37]

Phthalimide potassium salt (5.27g, 28.5 mmol, Tokyo Chemical Industry Co., Ltd) were added to a DMF (80 mL) solution dissolving 2,6-bis(chloromethyl)pyridine (2.28 g, 13.0 mmol, Tokyo Chemical Industry Co., Ltd) under stirring at 130 °C for 3 h. Water (120 mL) was poured into the colorless suspension after cooling to RT and the suspension was filtered. The residue was dried under vacuum to obtain a white solid (4.83 g, 12.2 mmol, 94% yield).

^{1}H NMR (δ/ppm vs. TMS, DMSO-d_6) 7.77-7.75 (m, 5H, Ph-H, Py-H), 7.62-7.60 (m, 4H, Ph-H), 7.30 (d, 2H, Py−H), 4.78 (s, 4H, Py−CH_2−N).

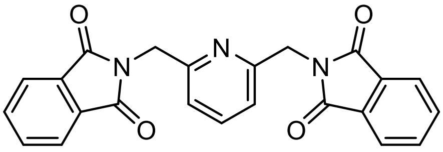

P1

P2.[37]

P1 (3.66 g, 9.22 mmol) and hydrazine monohydrate (4.48 mL, 92.1 mmol, Kanto Chemical Co., Inc.) were added to the EtOH (200 mL) under vigorously stirring at 70 °C for 3 h. The suspension was filtered after cooling to 0 °C. The filtrate was evaporated and poured into $CHCl_3$. The precipitate was further filtrated. The filtrate was evaporated and dried under vacuum to obtain a pale yellow oil (1.20 g, 8.72 mmol, 95% yield).

^{1}H NMR (δ/ppm vs. TMS, $CDCl_3$) 7.61 (t, 1H, Py−H), 7.13 (d, 2H, Py−H), 3.96 (s, 4H, Py−CH_2−N).

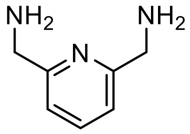

P2

H_2saldamp.

Salicylaldeyde (160 μL, 1.53 mmol, Tokyo Chemical Industry Co., Ltd) were added to a THF(10 mL) solution dissolving **P2** (105 mg, 0.765 mmol) under vigorous stirring at RT for 30 min, and then $NaBH(OAc)_3$ (496 mg, 2.34 mmol, Wako Pure Chemical Industries, Ltd.) were loaded portionwise at 0°C. The yellow suspension was stirred at 0 °C for 30 min, and then further agitated at RT for 2 h. Water was poured into the nearly colorless suspension to quench the reaction, followed by additional stirring for 15 min. After removal of THF by evaporation, 7.2 M NaOH aq was dropwise added to this aqueous mixture to make its pH 8-9, where oily layer was separated from the aqueous phase. This oily material was extracted by CH_2Cl_2 (50 mL), washed with a based brine (50 mL), and dried over $MgSO_4$ (Wako Pure Chemical Industries, Ltd.). This filtrate was evaporated to obtain a pale-yellow oil (229 mg, 0.655 mmol, 86% yield).

^{1}H NMR (δ/ppm vs. TMS, $CDCl_3$) 7.61 (t, 1H, Py−H), 7.17 (td, 2H, Ph−H), 7.09 (d, 2H, Py−H), 6.96 (dd, 2H, Ph−H), 6.84 (dd, 2H, Ph−H), 6.77 (dd, 2H, Ph−H), 4.00 (s, 4H, Py−CH_2−N), 3.93 (s, 4H, N−CH_2−Ph). ^{13}C NMR (δ/ppm vs. TMS, $CDCl_3$) 158.31, 157.72, 137.42, 128.91, 128.67, 122.53, 121.42, 119.17, 116.56, 53.02, 51.97. IR (v/cm^{-1} diamond ATR) 1086 ($\nu_{C\text{-}N}$).

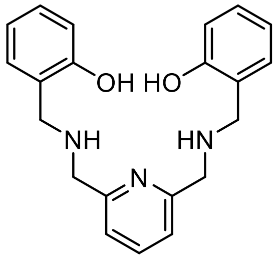

H_2saldamp

H_2saldiphan.

P3.[38] *o*-nitrofluorobenzene (1.87 mL, 2.50 g, 17.8 mmol, Tokyo Chemical Industry Co., Ltd) were added to DMSO (50 mL) solution dissolving *o*-nitroaniline (2.46 g, 17.8 mmol Tokyo Chemical Industry Co., Ltd) and K_2CO_3 (3.06 g, 22.1 mmol, Wako Pure Chemical Industries, Ltd.) under stirring at 120 °C for 48 h. After cooling to room temperature, 25 mL of water were added to the solution. The solution was extracted by CH_2Cl_2 (50 mL), washed with a brine (50 mL), and dried over $MgSO_4$ (Wako Pure Chemical Industries, Ltd.). This filtrate was evaporated to obtain a red solid (4.50 g, 17.4 mmol, 98% yield).

^{1}H NMR (δ/ppm vs. TMS, $CDCl_3$) 11.01 (br s, 1H, NH), 8.21 (dd, 2H, Ph−H), 7.60-7.51 (m, 4H, Ph−H), 7.10 (td, 2H, Ph−H).

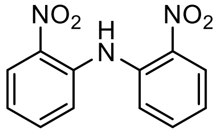

P3

P4.[39] Zinc powder (3.54 g, 54.1 mmol, Wako Pure Chemical Industries Ltd.) and NH_4Cl (2.68 g, 50.2 mmol, Wako Pure Chemical Industries, Ltd.) were added to the THF (50 mL) solution dissolving **P3** (0.999 g, 3.85 mmol) and refluxed at 70 °C for 19 h. After cooling to room temperature, the suspension was filtrate. The filtrate was evaporated and extracted with CH_2Cl_2 (50 mL). The organic layer was washed with brine (50 mL) and dried over $MgSO_4$ (Wako Pure Chemical Industries, Ltd.). This filtrate was evaporated to obtain a brown oil (0.697 g, 3.50 mmol, 81% yield).

^{1}H NMR (δ/ppm vs. TMS, $CDCl_3$) 6.92-6.87 (m, 2H, Ph−H), 6.80-6.70 (m, 6H, Ph−H), 5.00 (br s, 1H, NH), 3.59 (br s, 4H, NH).

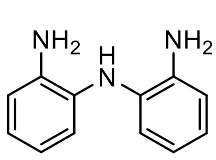

P4

P5. Salicylaldehyde (643 μL, 6.17 mmol, Tokyo Chemical Industry Co., Ltd) were added to a THF (20 mL) solution dissolving **P4** (0.614 g, 3.08 mmol) under stirring at 70 °C for 2 h. After evaporation of THF, the

residue was washed with 2-propanol to obtain a yellow solid (0.804 g, 1.97 mmol, 64% yield).
^{1}H NMR (δ/ppm vs. TMS, $CDCl_3$) 12.67 (s, 2H, OH), 8.62 (s, 2H, N=CH−Ph), 7.42-7.37 (m, 4H, Ph−H), 7.29-7.19 (m, 4H, Ph−H), 7.14 (d, 2H, Ph−H), 6.97 (t, 2H, Ph−H), 6.90 (t, 2H, Ph−H), 6.73 (d, 2H, Ph−H), 6.62 (br s, 1H, NH).

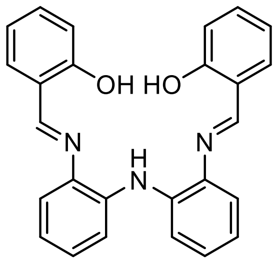

P5

H_2saldiphan. $NaBH(OAc)_3$ (1.26 g, 5.93 mmol Wako Pure Chemical Industries, Ltd.) were added to the THF (20 mL) solution dissolving **P5** (0.404 g, 0.992 mmol) at 0 °C under vigorous stirring. The yellow suspension was stirred at 0 °C for 30 min, and then further agitated at RT for 2 h. Water was poured into the nearly colorless suspension to quench the reaction, followed by additional stirring for 15 min. After removal of THF by evaporation, 7.2 M NaOH aq was dropwise added to this aqueous mixture to make its pH 8-9, where oily layer was separated from the aqueous phase. This oily material was extracted by CH_2Cl_2 (50 mL), washed with a based brine (50 mL), and dried over $MgSO_4$ (Wako Pure Chemical Industries, Ltd.). This filtrate was evaporated to obtain a yellow oil (0.386 g, 0.938 mmol, 95% yield).
^{1}H NMR (δ/ppm vs. TMS, $CDCl_3$) 7.18 (td, 2H, Ph−H), 7.14 (dd, 2H, Ph−H), 7.01 (dd, 2H, Ph−H), 6.92 (dd, 2H, Ph−H), 6.85 (m, 6H, Ph−H), 6.74 (dd, 2H, Ph−H), 4.96 (br s, 1H, NH), 4.37 (s, 4H, N−CH_2−Ph). ^{13}C NMR (δ/ppm vs. TMS, $CDCl_3$) 139.67, 132.60, 129.30, 129.05, 124.25, 123.18, 121.37, 120.49, 120.29, 116.68, 114.78, 48.33.

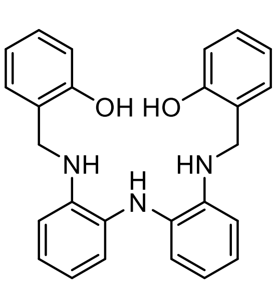

H_2saldiphan

H_2salphenazine.
P6.[40] Phenazine (1.81 g, 10.0 mmol, Tokyo Chemical Industry Co., Ltd) were added to the mixed solution of concentrated sulfuric acid (27 mL Wako Pure Chemical Industries, Ltd.) and fuming nitric acid (18 mL Wako Pure Chemical Industries, Ltd.) and stirred at 110 °C for 2.5 h. After cooling to room temperature,

the solution was added to 1 kg of ice. The precipitate solid was filtrated and washed with water and heated ethanol. The product was recrystallized with acetone and filtrate was collected to separate structural isomers (1,6-dinitrophenazine). The filtrate was evaporated and further recrystallized with acetic anhydride two times to obtain a yellow needle crystal (0.558 g, 2.07 mmol, 20% yield).

^{1}H NMR (δ/ppm vs. TMS, $CDCl_3$) 8.53 (dd, 2H, Ph−H), 8.38 (dd, 2H, Ph−H), 8.02 (m, 2H, Ph−H).

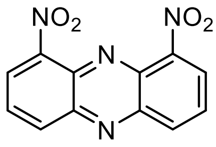

P6

P7.[40] 10% Pd/C (0.100 g, N.E. Chemcat Co.) were added to the Schlenk flask containing **P6** (0.537 g, 1.99 mmol). After the flask was purged with argon, EtOH (90 mL) was added. The suspension was stirred under hydrogen atmosphere at ambient temperature for 21 h. The reaction mixture was filtered with celite. The filtrate was evaporated to obtain a purple solid (0.303 g, 1.44 mmol, 72% yield).

^{1}H NMR (δ/ppm vs. TMS, $CDCl_3$) 7.62 (d, 2H, Ph−H), 7.57 (m, 2H, Ph−H), 6.93 (dd, 2H, Ph−H).

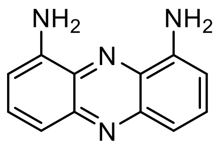

P7

P8. Salicylaldehyde (309 μL, 0.361 g, 2.96 mmol, Tokyo Chemical Industry Co., Ltd.) and AcOH (1.0 mL) were added to a THF (30 mL) dissolving **P7** (0.311 g, 1.48 mmol). The solution was stirred at 70 °C for 3 h. After removal of solvent, precipitated solid were washed with 2-propanol. Then the filter cake was dried in vacuum to obtain a brown solid (0.483 g, 1.15 mmol, 78% yield).

^{1}H NMR (δ/ppm vs. TMS, $CDCl_3$) 9.23 (s, 2H, N=CH−Ph), 8.14 (dd, 2H, Ph−H), 7.90 (m, 2H, Ph−H), 7.63 (dd, 2H, Ph−H), 7.34 (td, 2H, Ph−H), 7.28 (dd, 2H, Ph−H), 6.94 (d, 2H, Ph−H), 6.77 (td, 2H, Ph−H).

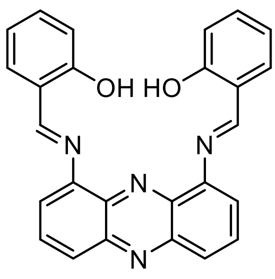

P8

H_2salphenazine. $NaBH(OAc)_3$ (0.228 g, 1.08 mmol, Wako Pure Chemical Industries, Ltd.) were loaded

portionwise to a THF (10 mL) dissolving **P8** (0.069 g, 0.17 mmol) under vigorous stirring at 0 °C for 30 min and then further agitated at RT for 2 h. Water was poured into the suspension to quench the reaction, followed by additional stirring for 15 min. After removal of THF by evaporation, 7.2 M NaOH aq was dropwise added to this aqueous mixture to make its pH 8-9, where oily layer was separated from the aqueous phase. This oily material was extracted by CH_2Cl_2 (20 mL), washed with a based brine (20 mL), and dried over $MgSO_4$ (Wako Pure Chemical Industries, Ltd.). This filtrate was evaporated to obtain a purple solid (0.062 g, 0.15 mmol, 89% yield).

^{1}H NMR (δ/ppm vs. TMS, $CDCl_3$) 7.66-7.55 (m, 4H, Ph−H), 7.32-7.23 (m, 4H, Ph−H), 6.97-6.83 (m, 6H, Ph−H), 4.68-4.64 (m, 4H, NH−CH_2−Ph).

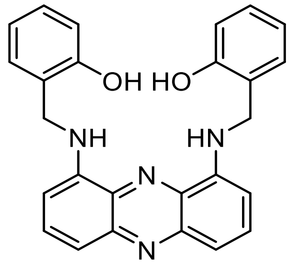

H_2salphenazine

Synthesis of Metal Complexes.

Caution! *^{238}U is an alpha emitter, and therefore standard precautions for handling radioactive materials should be followed.*

UO_2(saldamp). H_2saldamp (0.283 g, 0.809 mmol) were dissolved in THF (3 mL), with vigorous agitation, $UO_2(NO_3)_2{\cdot}6H_2O$ (0.447 g, 0.890 mmol) dissolved in ethanol (500 μL) and 7.2 M triethylamine (225 μL, 1.62 mmol, Wako Pure Chemical Industries, Ltd.) were dropwise added to this solution. After evaporation, the reddish oil was extracted with CH_2Cl_2 (20 mL), and evaporated to give reddish solid of UO_2(saldamp) (0.223 g, 0.361 mmol, 46% yield). Recrystallization from DMSO and water afforded block crystals of UO_2(saldamp) suitable for SCXRD.

Characterization of UO_2(saldamp). Crystallographic data for UO_2(saldamp) (CCDC 2498008): $C_{21}H_{21}N_3O_4U$, F_w = 617.44, 0.07 × 0.09 × 0.09 mm^3, monoclinic, $C2/c$ (No. 15), a = 11.2929(3) Å, b = 9.98911(18) Å, c = 17.7066(3) Å, β = 90.339(2)°, V = 1997.01(7) Å^3, Z = 4, T = 293(2) K, D_{calcd} = 2.054 g cm^{-3}, μ = 8.161 cm^{-1}, GOF = 1.031, R ($I > 2\sigma$) = 0.0216, wR (all) = 0.0460. ^{1}H NMR (δ/ppm vs. TMS, $CDCl_3$) 8.21 (t, 1H, Py-H), 7.87 (d, 2H, Py-H), 7.35 (dd, 2H, Ph-H), 7.29 (td, 2H, Ph-H), 6.89 (dd, 2H, Ph-H), 6.56 (tt, 2H, NH) ,6.51 (td, 2H, Ph-H), 5.15 (dd, 2H, equatrial), 4.83 (t, 2H, axial), 4.70 (t, 2H, axial), 4.53 (dd, 2H, equatrial).^{13}C NMR (δ/ppm vs. TMS, DMSO-d_6) 168.79, 160.96, 140.95, 129.41, 129.29, 127.46, 121.72, 119.27, 116.35, 58.20, 54.50. IR (ν/cm^{-1} diamond ATR) 3230 (ν_{N-H}),1268 (ν_{C-N}), 872 (ν_3 of UO_2^{2+}).

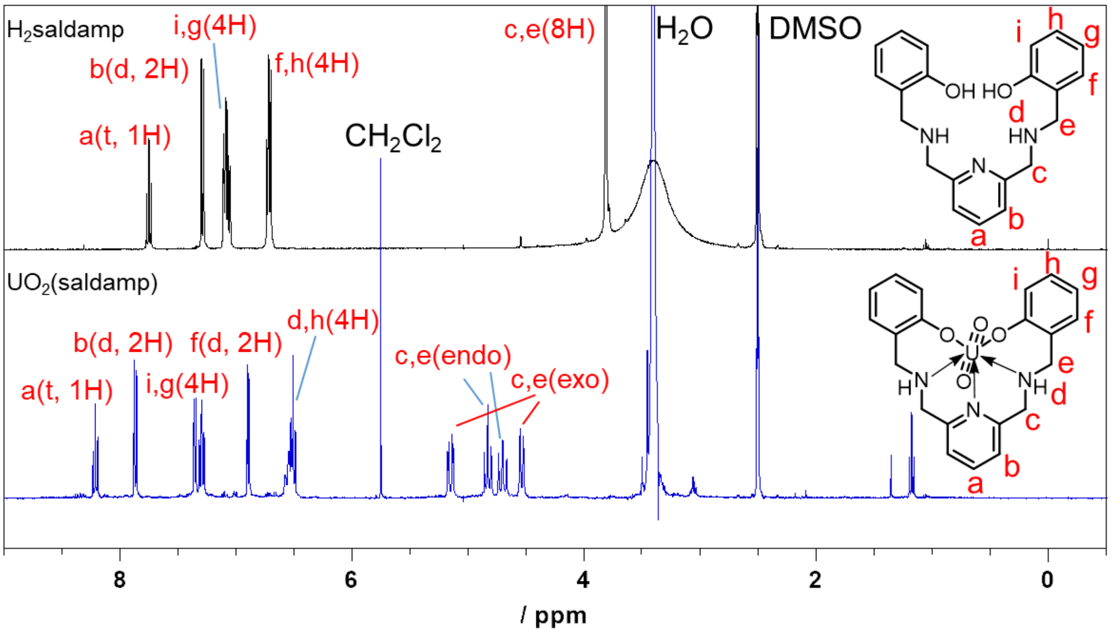

Figure S1. ^{1}H NMR spectra of H_2saldamp (black) and UO_2(saldamp) (blue) in DMSO-d_6.

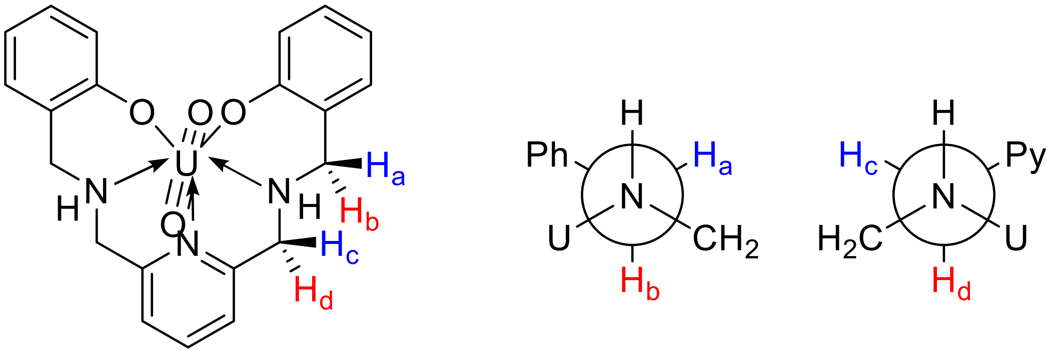

Figure S2. Molecular structure of UO_2(saldamp) and Newman projections along C−N bonds of UO_2(saldamp). Red : *endo* H, Blue : *exo* H.

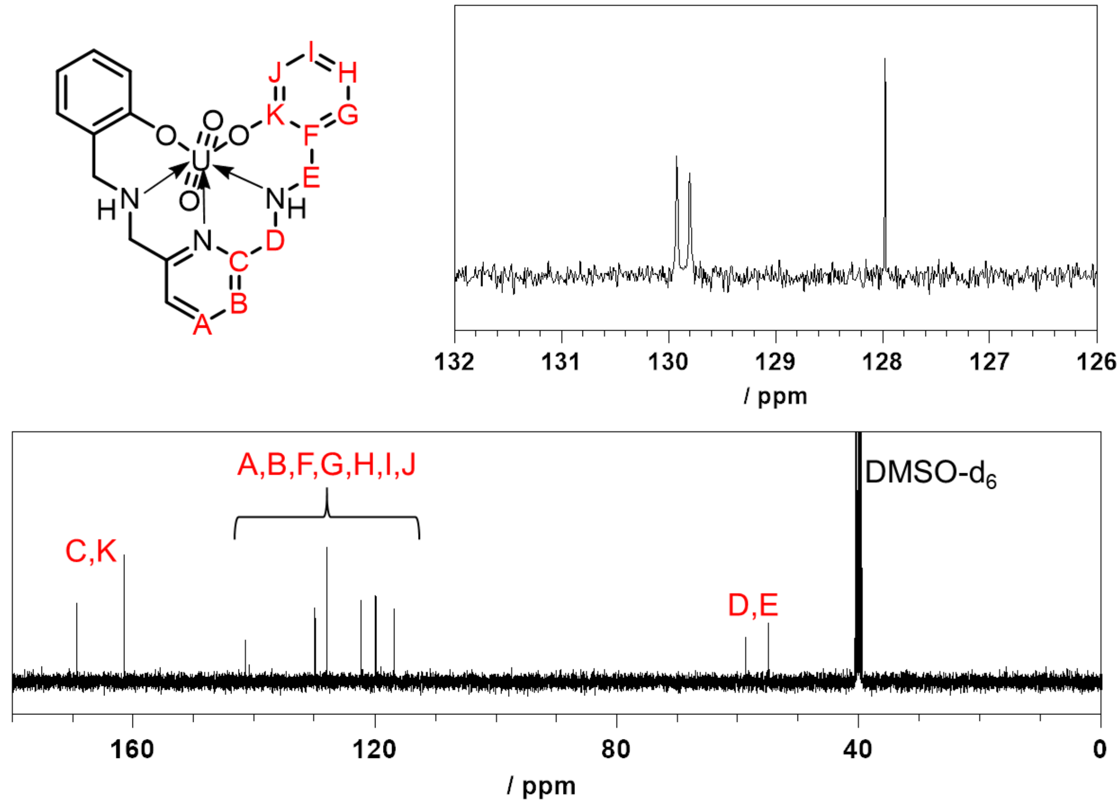

Figure S3. ^{13}C NMR spectrum of UO_2(saldamp) in DMSO-d_6.

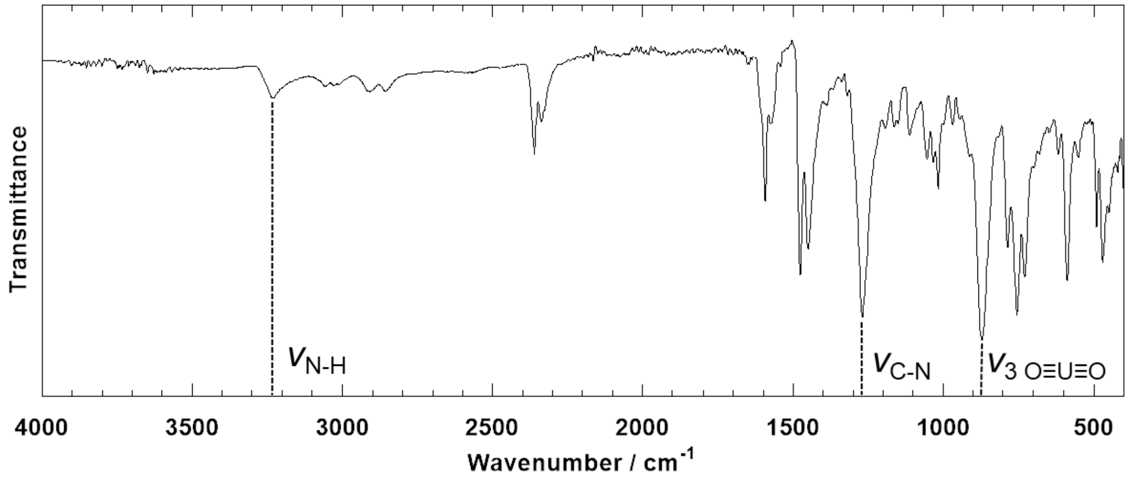

Figure S4. IR spectrum of UO_2(saldamp).

$CuCl_2$(H_2saldamp). $CuCl_2 \cdot 2H_2O$ (10.7 mg, 0.0628 mmol, Wako Pure Chemical Industries, Ltd.) were added to a EtOH (1.0 mL) solution dissolving H_2saldamp (19.8 mg, 0.0566 mmol). The blue solution was evaporated slowly at RT under an ambient atmosphere to obtain blue platelet crystals suitable for X-ray crystallography.

Characterization of $CuCl_2$(H_2saldamp). Crystallographic data for $CuCl_2$(H_2saldamp) (CCDC 2498009):

$C_{21}H_{23}Cl_2CuN_3O_2$, F_w = 483.88, 0.070 × 0.0230 × 0.0250 mm^3, monoclinic, $P2_1/c$ (No. 14), a = 16.543(2) Å, b = 9.2829(10) Å, c = 15.4072(18) Å, β = 111.927(8)°, V = 2194.9(5) Å^3, Z = 4, T = 296 K, D_{calcd} = 1.458 g cm^{-3}, μ = 1.260 cm^{-1}, GOF = 1.050, R ($I > 2\sigma$) = 0.0980, wR (all) = 0.2935.

Methods.

Characterization of Ligands and Uranyl Complexes.

^{1}H NMR and ^{13}C NMR spectra were recorded by JEOL JNM ECX-400 (^{1}H: 399.78 MHz). IR spectra were measured by JASCO FT/IR-4700 equipped with a diamond ATR apparatus.

The structural characterization of UO_2(saldamp) and Cu(H_2saldamp) have been carried out by single crystal X-ray diffraction. A single crystal of UO_2(saldamp) or Cu(H_2saldamp) was mounted on a Kapton capillary and located in the cryogenic N_2 stream at the specified temperature. Intensity data were collected using Rigaku XtaLab mini II or Rigaku RAXIS RAPID with graphite monochromated Mo-Kα radiation (λ = 0.71075 Å). The obtained diffraction data were analysed by Olex2[41] or CrysralStructure[42] software package suited with SHELX.[43,44] The structure was solved by SHELXT[44] or SIR92[45], and expanded using Fourier techniques. All non-hydrogen atoms were anisotropically refined by SHELXL 2018/3 or 2014/7.[46] Hydrogen atoms were refined as riding on their parent atoms with U_{iso}(H) = 1.2 U_{eq}(C, N). The final cycle of the full-matrix least-squares refinement of F^2 was based on the observed reflections and parameters, and converged with the unweighted and weighted agreement factors, R and wR, respectively. Crystallographic data of UO_2(saldamp) and $CuCl_2$(H_2saldamp) were summarized in the synthetic parts described above. CCDC 2498008 (UO_2(saldamp)) and 2498009 ($CuCl_2$(H_2saldamp)) contain the supplementary crystallographic data for this paper. These data can be obtained free of charge via www.ccdc.cam.ac.jk/data_request/cif, or by emailing data_request@ccdc.cam.ac.uk, or by contacting The Cambridge Crystallographic Data Centre, 12 Union Road, Cambridge CB2 1EZ, UK; fax: +44 1223 336033.

UV-vis Titration.

All titration experiments have been performed under a simulated seawater condition, which consists of 0.50 M NaCl + 2.3 mM HCO_3^-/CO_3^{2-} at pH 8.0, if not specified. During the whole titration experiment, temperature of the sample solution was kept at 298 K in a thermostat cell holder equipped with the spectrophotometer. To determine overall protonation constants (log β_{nH}) of saldamp^{2-}, we performed the spectrophotometric titration experiments in 0.50 M NaCl aq without any carbonate and bicarbonate. Stock solutions of H_2saldamp (10 mM) in EtOH (25 μL) was added to the 0.50 M NaCl aq (2.50 mL) in the quartz cuvette to prepare the initial sample solution in the titration experiments. The pH value of the sample solution was stepwise varied by adding NaOH aq or HCl aq, and the UV-vis absorption spectrum at each step was recorded by the Agilent Cary 3500 spectrophotometer. The collected spectra through pH variation were analyzed by HypSpec[15], where the first to fifth protonation equilibria of saldamp^{2-} species have been considered to give $H_n$saldamp^{n-2} (n = 1-5).

About UO_2^{2+}, a stability constant of UO_2(saldamp) was hardly determined by the stepwise addition of stock solution of UO_2^{2+} (8.33 mM) in water to the simulated seawater (2.50 mL) dissolving saldamp^{2-} (0.10

mM) in the quartz cuvette because of the too strong coordination of saldamp^{2-} to UO_2^{2+} as discussed in the main text. The stability constant was instead determined by UV-vis titration with pH variation under absence of HCO_3^-/CO_3^{2-}. Stock solutions of H_2saldamp (10 mM) in EtOH (5.0 μL) and UO_2^{2+} (5.06 mM) in water (9.9 μL) were added to the 0.50 M NaCl aq (2.50 mL) in the quartz cuvette. The pH value of the sample solution was stepwise varied by adding NaOH aq or HCl aq, and the UV-vis absorption spectrum at each pH was recorded by the Agilent Cary 3500 spectrophotometer. The absorption spectra collected through the pH variation were analyzed by HypSpec[15] to determine a stability constant, log β_U, of UO_2(saldamp).

To determine the specific selectivity of saldamp^{2-} towards UO_2^{2+}, stability constants of other metal ions were measured. The stock solution of H_2saldamp in EtOH (25 μL) was added to the simulated seawater (2.50 mL) in the quartz cuvette to prepare the initial sample solution in the titration experiments. The total concentration of a metal ion (M^{n+}) was stepwise increased by adding its feed solution (8.33 mM, M^{n+} = Al^{3+}, VO_2^+, Ni^{2+}, Cu^{2+}, Zn^{2+}) prepared by dissolving a chloride salt of M^{n+} in the simulated seawater. The UV-vis absorption spectrum at each increment step was recorded by Agilent Cary 3500 spectrophotometer. The obtained titration series of the UV-vis absorption spectra was analyzed by HypSpec (version 1.1.33)[15] to determine a stability constant of a metal complex with the saldamp^{2-}. For simplicity, only M:L^{2-} = 1:1 (L^{2-} = saldamp^{2-}) was considered in this analysis. This treatment should be valid, because each spectral series shows isosbestic points, or varies monotonously. Indeed, all the collected spectral series were well reproduced on this assumption. In the UV-vis titration experiments studied here, precipitation was not observed under any conditions.

DFT Calculations.

Quantum chemical calculations were performed using the Gaussian 16 program (Gaussian Inc.) rev.B01[47] employing density functional theory (DFT) by using a conductor like polarizable continuum model.[48] Structure optimizations were performed at the B3LYP level[49,50] with empirical dispersion corrections by Grimme and coauthors[51] followed by vibrational frequency analysis at the same level to confirm that there is no imaginary frequency present. The energy consistent small-core effective core potential (ECP) and the corresponding basis set suggested by Küchle et al. were used for U,[52] whereas correlation consistent cc-pVTZ basis sets were employed on C,O and H.[53] The spin–orbit effects and basis set superposition error corrections were neglected.

Time-dependent DFT (TD-DFT) calculations were performed using the ORCA program (version 6.0.1)[54,55] based on the DFT-optimized structures obtained from the Gaussian 16 calculations and using the same DFT functionals as well as the same basis sets and effective core potential (ECP). Theoretical electronic-transition spectra were recorded for 200 excited states.

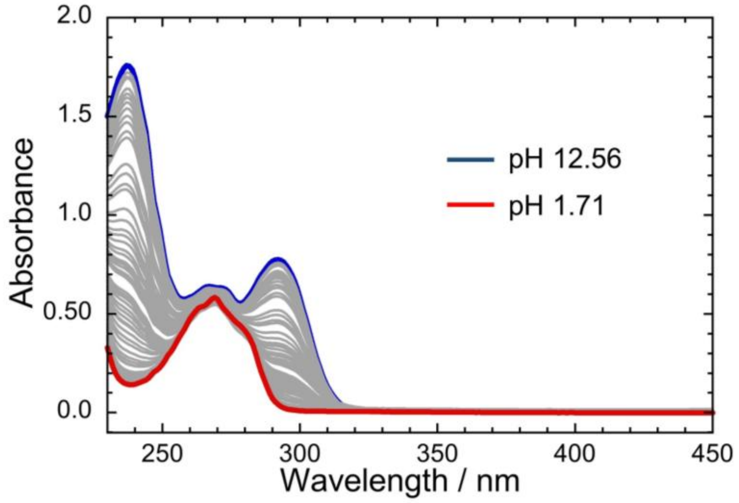

Figure S5. UV-vis absorption spectra of H_2saldamp (1.0×10^{-4} M) at different pH in 0.50 M NaCl + 2.3 mM HCO_3^-/CO_3^{2-} aqueous solution at 298 K.

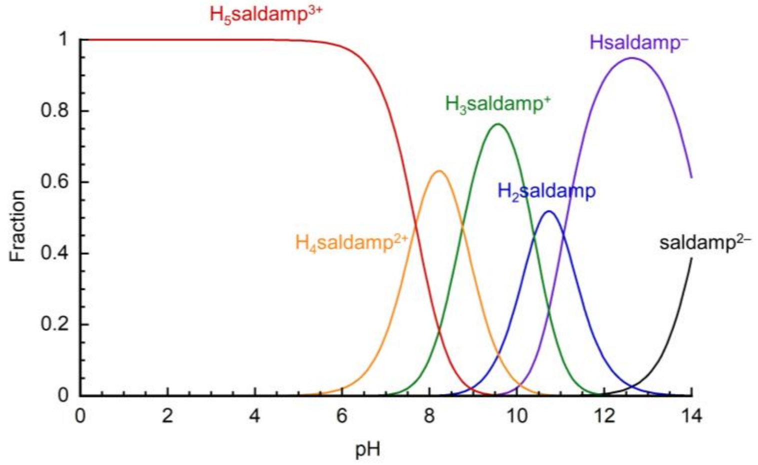

Figure S6. Speciation diagram of saldamp^{2-} species (0.10 mM) at 0.50 M NaCl + 2.3 mM HCO_3^-/CO_3^{2-} and 298 K.

Table S1. Selected bond lengths (Å) of UO_2(saldamp) and UO_2(saldian).

	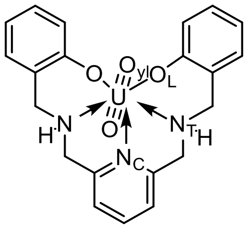 UO_2(saldamp)	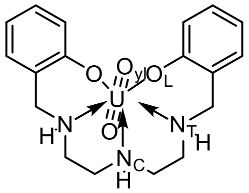 UO_2(saldian)
U−O_{yl}	U1≡O1 = 1.787(2)	U1≡O1 = 1.798(2)<br>U1≡O2 = 1.792(2)
U−O_L	U1–O2 = 2.225(2)	U1–O3 = 2.230(2)<br>U1–O4 = 2.215(2)
U−N_T	U1–N1 = 2.616(2)	U1–N1 = 2.613(3)<br>U1–N3 = 2.650(3)
U−N_C	U1–N2 = 2.591(3)	U1–N2 = 2.574(3)
ref.	This work	[14]

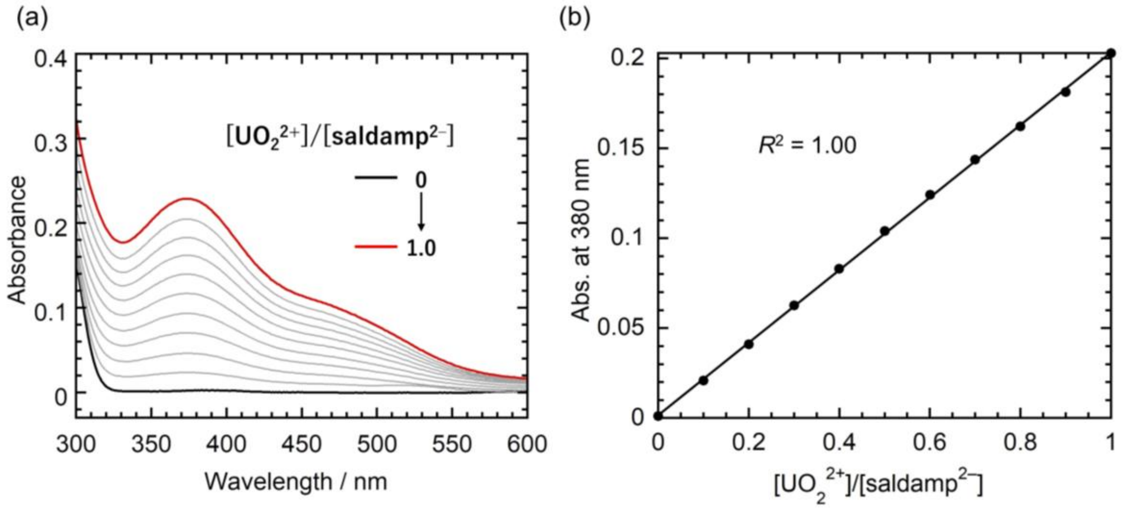

Figure S7. (a) UV-vis absorption spectra of aqueous solution of H_2saldamp (1.0×10^{-4} M) at different total UO_2^{2+} concentrations under the simulated seawater condition (0.50 M NaCl +2.3 mM HCO_3^-/CO_3^{2-}, pH 8, 298 K) and (b) absorbance at 380 nm in panel (a).

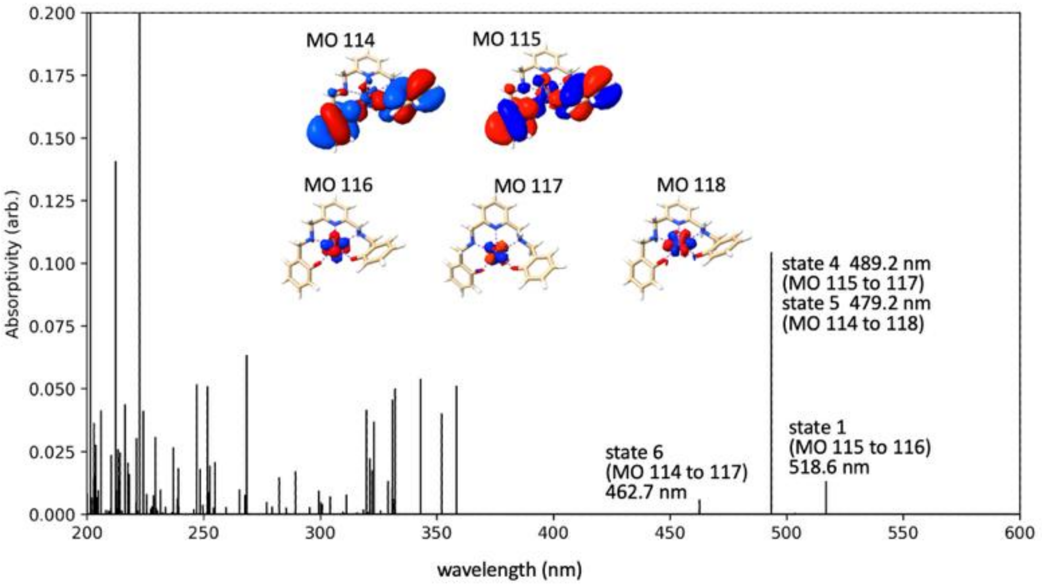

Figure S8. Predicted vertical transitions (vertical black lines) of the UO_2(saldamp) complex and the key excited states and molecular orbitals (MO 114 to MO118) involved.

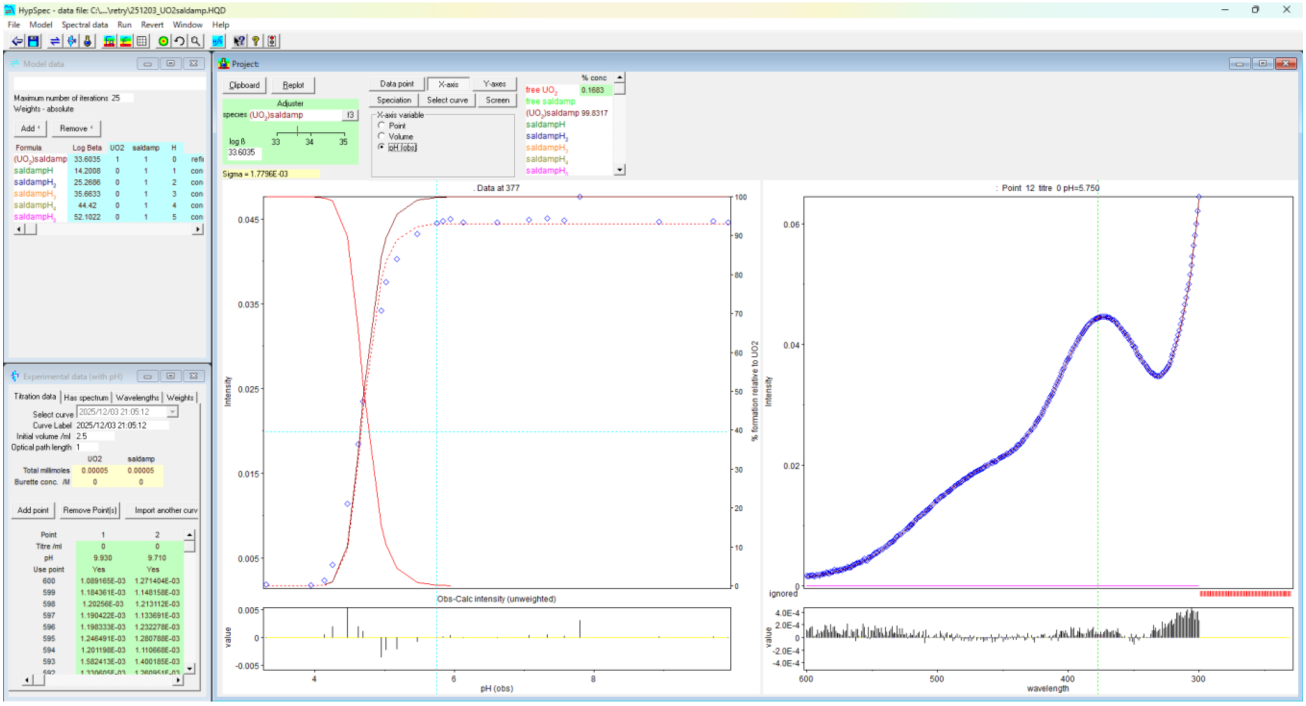

Figure S9. A screenshot of the HypSpec analysis of log β_U of UO_2(saldamp).

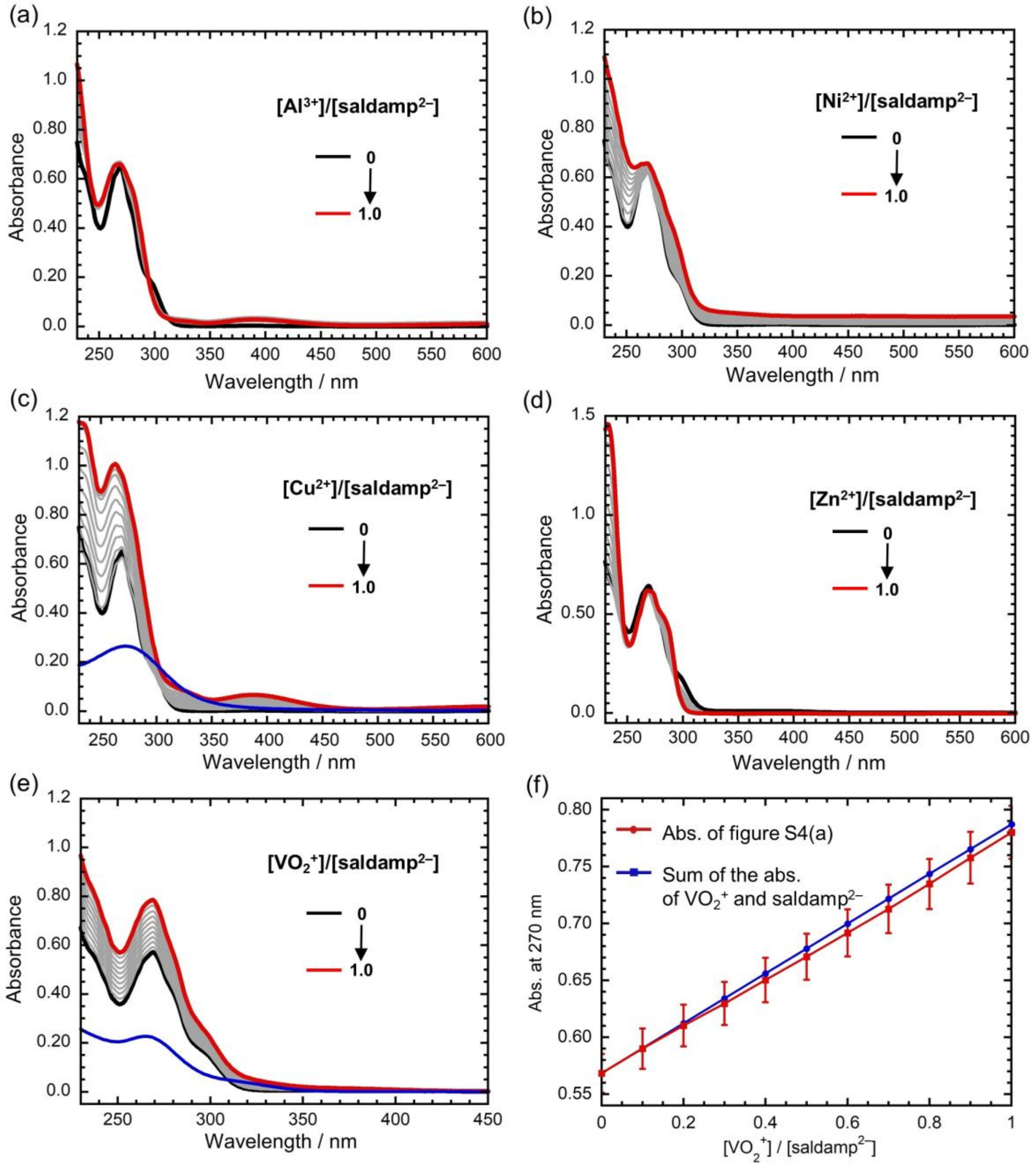

Figure S10. UV-vis absorption spectra of aqueous solutions of H_2saldamp (1.0×10^{-4} M) at different total concentrations of selected metal ions (M's) under the simulated seawater condition (0.50 M NaCl + 2.3 mM HCO_3^- /CO_3^{2-}, pH 8, 298 K). M = Al^{3+} (a), Ni^{2+} (b), Cu^{2+} (c), Zn^{2+} (d), and VO_2^+ (e). Blue lines indicate original absorption spectra of M's at 0.10 mM, if there is significant absorption. (f) Absorbance at 270 nm in panel (e) (red line) together with sum of the absorbance of VO_2^+ and saldamp^{2-} at a specified [VO_2^+]/[saldamp^{2-}] (blue line). Error bars represent estimated experimental errors based on uncertainty in actual pipetting operations.

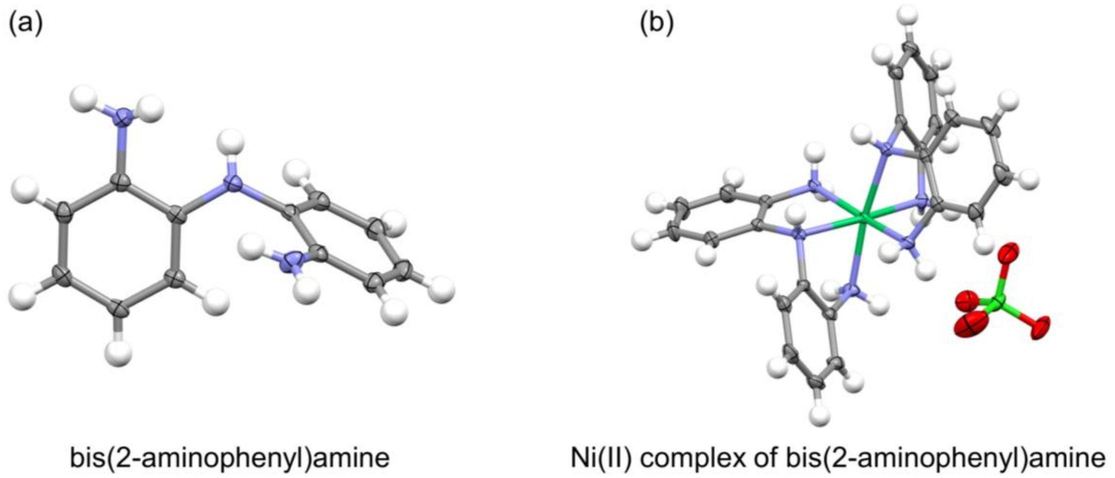

Figure S11. Molecular structures of free form of bis(2-aminophenyl)amine[35] (a), its Ni(**II**) complex[36] (b) reported elsewhere.

Cartesian Coordinates of DFT-Optimized Structures

UO_2(saldian) E = -1642.70183741 hartree

C -2.396257 2.837829 -0.121897

H -2.427221 3.027154 -1.196642

H -3.270183 3.324868 0.319380

C 1.970928 2.580333 1.274033

H 1.303337 2.560272 2.135435

H 2.912827 3.036637 1.593324

N -2.435772 1.377551 0.080343

H -2.585927 1.203395 1.070260

N 2.169758 1.182846 0.840545

H 2.303914 0.617383 1.672088

C 3.348196 0.962693 -0.041513

H 4.165398 1.626828 0.252165

H 3.063187 1.234159 -1.059709

C -3.553924 0.732641 -0.659833

H -4.461113 1.336355 -0.568781

H -3.267539 0.720146 -1.713492

C -3.826587 -0.660147 -0.165558

C -5.065078 -1.013906 0.362083

C -2.805797 -1.633252 -0.245572

C -5.322587 -2.309322 0.801241

H -5.841597 -0.261108 0.426152

C -3.069978 -2.936396 0.199651

C -4.316325 -3.267860 0.714304

H -6.292280 -2.565400 1.205206

H -2.280541 -3.673075 0.129998

H -4.501197 -4.279377 1.052894

C 3.828119 -0.465310 0.012666

C 2.914015 -1.539917 -0.100565

C 5.184756 -0.743022 0.162376

C 3.407895 -2.854285 -0.068258

C 5.666189 -2.047790 0.185838

H 5.878245 0.083681 0.259294

C 4.765624 -3.103218 0.068376

H 2.698323 -3.666463 -0.155079

H 6.724737 -2.236245 0.299457

H 5.121328 -4.125459 0.088091

O -1.617753 -1.308462 -0.748001

O 1.611332 -1.325003 -0.225163

U -0.038205 0.160637 -0.198262

O -0.368142 -0.046862 1.554068

O 0.269417 0.526759 -1.930796

N 0.038708 2.834071 -0.231884

H -0.083213 3.000455 -1.224951

C -1.123076 3.424298 0.460217

H -1.136828 4.512707 0.352494

H -1.048824 3.197682 1.524396

C 1.348631 3.400789 0.161581

H 1.249486 4.442947 0.474276

H 1.994388 3.386164 -0.715087

UO_2(saldamp) E = -1755.86763155 hartree

C 0.964272 3.101954 -0.644819

C 0.999398 4.489173 -0.658830

C -0.000168 5.190297 0.000011

C -0.999792 4.489169 0.658737

C -0.964777 3.101938 0.644537

N -0.000270 2.428502 -0.000162

H -0.000118 6.271191 0.000090

H 1.795294 5.004009 -1.176219

H -1.795664 5.003994 1.176175

C -1.989134 2.267686 1.358904

H -2.887808 2.857423 1.554981

H -1.584703 1.954805 2.324420

C 1.988506 2.267681 -1.359327

H 2.887078 2.857452 -1.555758

H 1.583816 1.954602 -2.324667

N -2.291349 1.048893 0.585905

H -2.689167 1.330108 -0.307086

N 2.291053 1.049025 -0.586199

H 2.689085 1.330425 0.306641

C 3.299097 0.198252 -1.277973

H 4.150772 0.807869 -1.591065

H 2.814446 -0.186326 -2.177470

C -3.299518 0.198116 1.277556

H -2.815126 -0.186207 2.177300

H -4.151390 0.807684 1.590202

C -3.769326 -0.932095 0.409427

C -5.100671 -1.063745 0.028268

C -2.825801 -1.893149 -0.014524

C -5.523547 -2.135007 -0.753749

H -5.816757 -0.318403 0.353085

C -3.257042 -2.970778 -0.800728

C -4.592984 -3.087272 -1.162284

H -6.562462 -2.224778 -1.039448

H -2.526614 -3.704042 -1.116102

H -4.908731 -3.925237 -1.770663

C 3.769412 -0.931727 -0.409791

C 2.826176 -1.892745 0.014903

C 5.100971 -1.063221 -0.029344

C 3.257920 -2.970155 0.801134

C 5.524351 -2.134281 0.752686

H 5.816835 -0.317911 -0.354724

C 4.594072 -3.086500 1.161961

H 2.527712 -3.703393 1.117085

H 6.563429 -2.223927 1.037829

H 4.910202 -3.924309 1.770356

O -1.552757 -1.761889 0.343827

O 1.552950 -1.761677 -0.342791

U -0.000049 -0.199392 0.000181

O 0.433373 -0.092085 1.738101

O -0.433580 -0.092783 -1.737757

UO_2(saldiphan) E: -1947.65113300 hartree

C -2.443491 1.689386 -0.936531

C 1.308953 1.945932 1.626614

N -2.386836 0.453767 -0.197241

H -2.367310 0.656029 0.799197

N 1.970758 0.850331 0.972127

H 2.310922 0.211269 1.682226

C 3.121042 1.204811 0.094542

H 3.734938 1.967976 0.576870

H 2.724527 1.652825 -0.819863

C -3.505724 -0.492638 -0.465362

H -4.463557 -0.028679 -0.226324

H -3.482592 -0.687255 -1.538561

C -3.401483 -1.790671 0.283423

C -4.394351 -2.190352 1.173230

C -2.319708 -2.656364 0.021749

C -4.347149 -3.432998 1.797941

H -5.220939 -1.519209 1.373419

C -2.282212 -3.911691 0.641576

C -3.287815 -4.293048 1.520194

H -5.127489 -3.726846 2.486201

H -1.449548 -4.567776 0.425481

H -3.240901 -5.264923 1.994590

C 3.984032 0.008836 -0.237981

C 3.456762 -1.295653 -0.388318

C 5.351242 0.207876 -0.428012

C 4.329442 -2.342129 -0.730675

C 6.204279 -0.831767 -0.776124

H 5.753787 1.205659 -0.300509

C 5.681908 -2.114003 -0.926770

H 3.909800 -3.333649 -0.836561

H 7.259579 -0.645520 -0.919014

H 6.330534 -2.938768 -1.192350

O -1.346036 -2.260619 -0.802873

O 2.169079 -1.564323 -0.190727

U 0.143749 -0.681867 -0.290174

O -0.232483 -0.945991 1.438825

O 0.433311 -0.274581 -2.011142

N -0.097191 2.019192 -0.329237

H 0.683028 2.201044 -0.952261

C -1.293882 2.484456 -0.986116

C 0.217185 2.534158 0.981091

C -1.276106 3.665593 -1.715812

C -3.567556 2.106959 -1.648666

C -2.401048 4.072904 -2.420922

H -2.379298 4.988239 -2.994991

C -3.544327 3.286984 -2.384235

H -4.428027 3.586161 -2.930636

C -0.522340 3.519296 1.619528

C 1.663150 2.365028 2.901342

C 0.921968 3.351782 3.544906

H 1.195279 3.664583 4.542793

C -0.170591 3.922122 2.905182

H -0.754457 4.684742 3.401322

H -4.469176 1.516653 -1.643658

H -0.370140 4.256850 -1.735969

H 2.506604 1.901209 3.395744

H -1.371625 3.963363 1.121915

UO_2(salphenazine) E(RB3LYP) = -2000.62297456

C 4.124616 3.542100 -0.185780

C 4.829972 2.380945 -0.267991

C 4.133645 1.145947 -0.367725

C 2.696543 1.151653 -0.412955

C 1.990103 2.395749 -0.389022

C 2.704881 3.554028 -0.245803

C 2.696163 -1.152322 -0.413655

C 4.133269 -1.147094 -0.368249

C 4.829205 -2.382343 -0.268909

H 5.908399 -2.359551 -0.231648

C 4.123480 -3.543319 -0.187326

C 2.703760 -3.554786 -0.247710

C 1.989346 -2.396228 -0.390553

H 4.641625 4.485787 -0.084223

H 5.909166 2.357822 -0.230957

H 2.202914 4.506518 -0.193051

H 4.640173 -4.487210 -0.086041

H 2.201484 -4.507148 -0.195535

N 0.566488 2.323114 -0.486441

H 0.312832 2.014285 -1.422046

N 0.565811 -2.323188 -0.488181

H 0.312254 -2.013660 -1.423563

C -0.175847 -3.573582 -0.174413

H 0.188757 -4.397436 -0.789537

H 0.046606 -3.804744 0.868453

C -0.174895 3.573561 -0.172147

H 0.190238 4.397676 -0.786594

H 0.047146 3.803985 0.870965

C -1.660107 -3.445825 -0.373831

C -2.386790 -2.508648 0.387864

C -2.340644 -4.275178 -1.260958

C -3.778758 -2.444085 0.251334

C -3.723383 -4.204609 -1.399503

H -1.777741 -4.990141 -1.848672

C -4.437771 -3.286719 -0.633986

H -4.320776 -1.719076 0.843646

H -4.234999 -4.857083 -2.093304

H -5.513827 -3.221579 -0.731228

C -1.659082 3.446345 -0.372486

C -2.386513 2.508909 0.388187

C -2.338796 4.276337 -1.259652

C -3.778404 2.444793 0.250670

C -3.721451 4.206178 -1.399205

H -1.775303 4.991439 -1.846629

C -4.436591 3.288067 -0.634658

H -4.321028 1.719598 0.842196

H -4.232416 4.859125 -2.093041

H -5.512594 3.223240 -0.732691

N 2.011447 -0.000220 -0.416909

N 4.816416 -0.000689 -0.352180

O -1.736819 1.688712 1.219920

O -1.736334 -1.689147 1.219668

U -0.440110 0.000041 0.605608

O 0.431688 0.000504 2.170204

O -1.193487 0.000182 -1.016748

References.

[14] T. Mizumachi, M. Sato, M. Kaneko, T. Takeyama, S. Tsushima, K. Takao, "Fully Chelating N_3O_2-Pentadentate Planar Ligands Designed for the Strongest and Selective Capture of Uranium from Seawater" *Inorg. Chem.* **2022**, *61*, 6175–6181.

[15] A. Sabatini, A. Vacca, P. Gans, "Mathematical algorithms and computer programs for the determination of equilibrium constants from potentiometric and spectrophotometric measurements" *Coord. Chem. Rev.* **1992**, *120*, 389–405.

[35] O. E. Palomero, R. A. Jones, "Accessing Pentagonal Bipyramidal Geometry with Pentadentate Pincer Amido-bis(amidate) Ligands in Group IV and V Early Transition Metal Complexes" *Organometallics* **2020**, *39*, 3689–3694.

[36] R. Sikari, S. Sinha, U. Jash, S. Das, P. Brandão, B. de Bruin, N. D. Paul, "Deprotonation Induced Ligand Oxidation in a Ni^{II} Complex of a Redox Noninnocent N^1-(2-Aminophenyl)benzene-1,2-diamine and Its Use in Catalytic Alcohol Oxidation" *Inorg. Chem.* **2016**, *55*, 6114–6123.

[37] G. Lee, M. Oka, H. Takemura, Y. Miyahara, N. Shimizu, T. Inazu, "Efficient Synthesis of 2,11,20-Triaza[3.3.3](2,6)pyridinophane" *J. Org. Chem.* **1996**, *61*, 8304–8306.

[38] P. Ren, O. Vechorkin, K. von Allmen, R. Scopelliti, X. Hu, "A Structure-Activity Study of Ni-Catalyzed Alkyl-Alkyl Kumada Coupling. Improved Catalysts for Coupling of Secondary Alkyl Halides" *J. Am. Chem. Soc.* **2011**, *133*, 7084–7095.

[39] R. F. Munhá, R. A. Zarkesh, A. F. Heyduk, "Tuning the Electronic and Steric Parameters of Redox-Active Tris(amido) Ligand" *Inorg. Chem.* **2013**, *52*, 11244–11255.

[40] E. Breitmaier, U. Hollstein, "Carbon-13 Nuclear Magnetic Resonance Chemical Shifts of Substituted Phenazines" *J. Org. Chem.* **1976**, *41*, 2104–2108.

[41] O. V. Dolomanov, L. J. Bourhis, R. J. Gildea, J. A. K. Howard, H. Puschmann, "*OLEX2*: a complete structure solution, refinement and analysis program" *J. Appl. Crystallogr.* **2009**, *42*, 339–341.

[42] CrystalStructure 4.2.7, Crystal Structure Analysis Package; Rigaku Corporation, Tokyo (Japan), **2017**.

[43] G. M. Sheldrick, "A short history of SHELX" *Acta Crystallogr.* **2008**, *A64*, 112–122.

[44] G. M. Sheldrick, "SHELXT-Integrated space-group and crystal-structure determination" *Acta Crystallogr.* **2015**, *A71*, 3–8.

[45] A. Altomare, G. Cascarano, C. Giacovazzo, A. Guagliardi, M. C. Burla, G. Polidori, M. Camalli, "*SIR92* – a program for automatic solution of crystal structures by direct methods" *J. Appl, Crystallogr.* **1994**, *27*, 435–436.

[46] G. M. Sheldrick, "Crystal structure refinement with SHELXL" *Acta Crystallogr.* **2015**, *C71*, 3–8.

[47] M. J. Frisch, G. W. Trucks, H. B. Schlegel, G. E. Scuseria, M. A. Robb, J. R. Cheeseman, G. Scalmani, V. Barone, G. A. Petersson, H. Nakatsuji, X. Li, M. Caricato, A. V. Marenich, J. Bloino, B. G. Janesko, R. Gomperts, B. Mennucci, H. P. Hratchian, J. V. Ortiz, A. F. Izmaylov, J. L. Sonnenberg, D. Williams-Young, F. Ding, F. Lipparini, F. Egidi, J. Goings, B. Peng, A. Petrone, T. Henderson, D. Ranasinghe, V. G. Zakrzewski, J. Gao, N. Rega, G. Zheng, W. Liang, M. Hada, M. Ehara, K. Toyota, R. Fukuda, J. Hasegawa, M. Ishida, T. Nakajima, Y. Honda, O. Kitao, H. Nakai, T. Vreven, K. Throssell, J. A. Montgomery Jr., J. E. Peralta, F. Ogliaro, M. J. Bearpark, J. J. Heyd, E. N. Brothers, K. N. Kudin, V. N. Staroverov, T. A. Keith, R. Kobayashi, J. Normand, K. Raghavachari, A. P. Rendell, J. C. Burant, S. S. Iyengar, J. Tomasi, M. Cossi, J. M. Millam, M. Klene, C. Adamo, R. Cammi, J. W.

Ochterski, R. L. Martin, K. Morokuma, O. Farkas, J. B. Foresman and D. J. Fox, Gaussian 16, Revision B.01, Gaussian, Inc., Wallingford CT (USA), **2016**.

[48] J. Tomasi, B. Mennucci and R. Cammi, "Quantum Mechanical Continuum Solvation Models" *Chem. Rev.* **2005**, *105*, 2999–3094.

[49] A. D. Becke, "Density-functional thermochemistry. III. The role of exact exchange" *J. Chem. Phys.* **1993**, *98*, 5648–5652.

[50] C. Lee, W. Yang and R. G. Parr, "Development of the Colle-Salvetti correlation-energy formula into a functional of the electron density" *Phys. Rev. B* **1988**, *37*, 785–789.

[51] S. Grimme, J. Antony, S. Ehrlich and H. Krieg "A consistent and accurate *ab initio* parametrization of density functional dispersion correction (DFT-D) for the 94 elements H-Pu" *J. Chem. Phys*. **2010**, *132*, 154104.

[52] W. Küchle, M. Dolg, H. Stoll and H. Preuss, "Energy-adjusted pseudopotentials for the actinides. Parameter sets and test calculations for thorium and thorium monoxide" *J. Chem. Phys.* **1994**, *100*, 7535–7542.

[53] T.H.Dunning, "Gaussian basis sets for use in corrected molecular calculations. I. The atoms boron through neon and hydrogen" *J. Chem. Phys.* **1989**, *90*, 1007–1023.

[54] F. Neese, "The ORCA program system" *Wiley Interdiscip. Rev.: Comput Mol. Sci.*, **2012**, *2*, 73–78.

[55] F. Neese, "Software update: the ORCA program system – Version 6.0" *Wiley Interdiscip. Rev.: Comput. Mol. Sci.*, **2025**, *15*, e70019.
